# Coexpression network and phenotypic analysis identify metabolic pathways associated with the effect of warming on grain yield components in wheat

**DOI:** 10.1371/journal.pone.0199434

**Published:** 2018-06-25

**Authors:** Christine Girousse, Jane Roche, Claire Guerin, Jacques Le Gouis, Sandrine Balzegue, Said Mouzeyar, Mohamed Fouad Bouzidi

**Affiliations:** 1 GDEC, Université Clermont Auvergne, INRA, Clermont–Ferrand, France; 2 Transcriptomic Platform of iPS2, INRA, Evry, France; Institute of Genetics and Developmental Biology Chinese Academy of Sciences, CHINA

## Abstract

Wheat grains are an important source of human food but current production amounts cannot meet world needs. Environmental conditions such as high temperature (above 30°C) could affect wheat production negatively. Plants from two wheat genotypes have been subjected to two growth temperature regimes. One set has been grown at an optimum daily mean temperature of 19°C while the second set of plants has been subjected to warming at 27°C from two to 13 days after anthesis (daa). While warming did not affect mean grain number per spike, it significantly reduced other yield-related indicators such as grain width, length, volume and maximal cell numbers in the endosperm. Whole genome expression analysis identified 6,258 and 5,220 genes, respectively, whose expression was affected by temperature in the two genotypes. Co-expression analysis using WGCNA (Weighted Gene Coexpression Network Analysis) uncovered modules (groups of co-expressed genes) associated with agronomic traits. In particular, modules enriched in genes related to nutrient reservoir and endopeptidase inhibitor activities were found to be positively associated with cell numbers in the endosperm. A hypothetical model pertaining to the effects of warming on gene expression and growth in wheat grain is proposed. Under moderately high temperature conditions, network analyses suggest a negative effect of the expression of genes related to seed storage proteins and starch biosynthesis on the grain size in wheat.

## Introduction

According to the fifth Assessment Report released by the Intergovernmental Panel on Climate Change (http://www.ipcc.ch/report/ar5/syr/), global surface temperature change, for the end of the 21st century, is projected to exceed 1.5°C to 2°C depending on the RCP scenario (Representative Concentration Pathways). The rise in the average global temperature will be associated with a higher frequency and a longer duration of heat waves on daily and seasonal timescales. These changes are likely to have negative effects on the yield of major crops such as bread wheat [1]. With an annual production reaching roughly 729 million tons in 2014 (http://faostat3.fao.org/browse/Q/QC/E), wheat is the third largest crop in the world, growing in many different environments and providing one fifth of the calories consumed by humans. In a global warming context, and as the extension of the wheat growing areas is limited, increased wheat productivity is needed to guarantee future food security [2, 3].

Wheat crops are sensitive to environmental variations and reduced grain yields are expected when crops are subjected to high temperatures during the reproductive phase, and, in particular, a few days before and after the anthesis [4, 5, 6]. For example, Lobell *et al*. [7] have quantified the impact of heat stress to a global loss of 5.5% in wheat yields, and a total loss of 35 million tons worldwide. In a recent study, Tack *et al*. [8] surveyed the performance of 268 wheat varieties over the years 1985–2013 in non-irrigated fields in Kansas, US. They found that warming causes an overall negative effect and that temperatures above 34°C during the springtime lead to the largest reductions in yield.

Several reviews discuss the effect of post-anthesis elevated temperatures on wheat grain yield [9, 10, 11, 12]. Depending on the timing, intensity and duration of their occurrence, high temperatures can reduce the number of grains per ear [13, 14] and/or the final grain dry mass [15, 16, 4]. The reduction in final grain mass generally results from a shortening of the grain filling period. In most cases, this reduction in the duration of grain filling is not fully compensated by an increase in the rate of grain dry matter accumulation [16, 17], depending on the genotype and on the degree of reduction in grain number per ear [18].

When subjected to post-anthesis high temperatures, alterations in the timing of grain development result from the different effects on the various processes occurring within the grain. A wheat grain consists of three main compartments: the outer layers, the endosperm and the embryo. The development of the wheat grain is classically divided into three phases [19]: i) the early or lag-phase (also named enlargement phase), ii) the filling-phase and iii) the maturation-desiccation phase. The first phase includes double fertilization of the ovule, giving rise to both the embryo and the endosperm. It is followed in the endosperm by rapid cellularization and cell proliferation [20]. During this phase, the dry mass of an individual grain increases slowly and the enlargement of the grain structure results mainly from the rapid influx of water into the endosperm. This phase is complete within about 15 daa depending on the wheat genotype and the environment. At the end of this phase, the grain length is set definitively, the grain water content has reached its maximum value, and cell proliferation ends in the endosperm [21, 22]. During the filling-phase (from ∼15 daa to ∼35 daa), cell expansion in the endosperm allows the grains to fill with starch and storage proteins and the grain dry mass increases linearly meanwhile the water mass of the grain remains steady (water plateau). At the end of this phase, the maximal grain dry mass is set and the grain has reached physiological maturity. During the last phase, protein and starch deposition ceases, the grain undergoes a period of maturation, rapidly desiccates and the endosperm tissue undergoes apoptosis.

The duration of the lag-phase associated with maximal endosperm cell number or that of the filling-phase associated with maximum dry matter or protein accumulation into the grain, can serve to identify specific stages of grain development. As suggested by Altenbach [23], these developmental markers that can be affected by high temperatures, can provide a framework for studying the molecular changes occurring during grain development in response to environmental variations.

The molecular and cellular response of plants to heat stress has been widely addressed in the literature. In particular, it is commonly found that heat stress induces the accumulation of Heat-Shock Proteins (HSPs) under the control of Heat Shock Factors (HSFs) [24]. In addition, several other cellular processes are affected by heat stress. For example, studies on rice found that heat responsive genes in panicles [25] and flag leaves [26] were mainly involved in transcriptional regulation, transport, protein binding and antioxidant.

In maturing tomato (*Solanum lycopersicum* L.) microspores, Frank *et al*. [27] found that HSPs, reactive oxygen species (ROS) scavengers, hormones, and sugars were involved in responses to heat stress. In *Brachypodium distachyon* L., two-week-old seedlings or eight-week-old plants were subjected to a heat stress at 42°C for one and for five hours. These treatments resulted in the alteration of 6,609 genes representing one-fourth of the gene content of *B*. *distachyon* [28]). The differentially expressed genes (DEGs) are involved in various pathways such as photosynthesis, protein folding and protein dephosphorylation. Similarly, 958 genes were induced and 1,122 others were repressed in barley caryopses exposed to 42°C [29]. As expected, the set corresponding to heat-induced genes was enriched in GO terms ‘response to stress’ and ‘response to biotic and abiotic’ stress.

In wheat, Qin *et al*. [30] identified a total of 6,560 probe sets that displayed changes in expression after heat treatment at 40°C with or without acclimatation at 34°C. This set included HSPs, HSFs, and gene encoding proteins involved in phytohormone biosynthesis/signaling, calcium, sugar signal pathways, RNA metabolism, ribosomal proteins, as well as primary and secondary metabolisms. Later, wheat tolerant genotype TAM107 was subjected to a severe heat stress (40°C for one and for six hours), drought stress or both of these combined [31]. They identified 29,395 differentially expressed genes in at least one stress condition compared to the control, among which 2,468 were affected only by heat stress (up- and down-regulated genes). However, they also found a large proportion of genes common to several stresses. In particular, analysis of GO terms enrichment indicated that some functional pathways were commonly regulated by drought stress, heat stress and heat plus drought stress. For instance, response to abiotic stress, response to hormones (ABA, JA, ethylene and GA) and carbohydrate metabolism were all enriched in commonly up-regulated genes, whereas GO terms related to photosynthesis were enriched in commonly down-regulated genes. Wan *et al*. [32] submitted wheat developing caryopses to moderately high temperature (28°C), to dry conditions as well as to dry and hot conditions finding that most environmental treatment effects on transcription were due to acceleration of development, but that a few transcripts were specifically affected.

While the effects of temperature are widely studied in plants, they are mainly addressed after a severe heat stress (*i*.*e* non-physiological temperatures) in leaves [33]. In contrast, the effects of a high, but physiological (not a heat shock), temperature on grain development in cereals are less documented.

In the present study, we surveyed a few agronomic traits during wheat grain development in response to low and moderately high temperatures called thereafter warming (19°C vs 27°C) in two genotypes. We also used NimbleGen microarray containing 40,656 UniGenes to characterize differentially expressed genes (DEGs). The results obtained suggest that warming (27°C), imposed from two to 13 daa induces an earlier expression of genes related to nutrients accumulation. In turn, the earlier onset of the nutrients accumulation process may be the signal for an accelerated development rate of the grain. The subsequent effect of these events is a net reduction in grain yield.

## Materials and methods

### Plant material and growth conditions

SxB49 and SxB139 are two *Triticum aestivum* L. recombinant inbred lines (RIL) derived from a cross between two elite spring wheat varieties; Seri M82 and Babax [34]. Briefly, Seri M82 is characterized by moderate tolerance to drought and high yield potential while Babax is a sister line of the elite variety Baviacora recognized for drought tolerance and high yield potential [34]. The two RIL were chosen for their similar phenology and because they have 92% of their alleles in common according to a SNP genotyping array [35]. Seeds from the two RILs were sown in Clermont-Ferrand, France (45°78' N, 3°08' E, 401 m a.s.l) 0.5m deep, in 2-m^2^ containers filled with a black peat soil. From sowing to anthesis, the crops were maintained under natural conditions. At anthesis, four selected containers were transferred to transparent enclosures under natural light in the Crop Climate Control and Gas Exchange Measurement (C3-GEM platform [36]). Except during high temperature treatment, air temperature was monitored and maintained at an average daily temperature of 19 ± 0.2°C (day/night temperature: 21/15°C). Day/night air relative humidity was maintained at 82.6 ± 7%. After ear emergence, 250 main stems with similar spikelet numbers (16.8 ± 1.0) were tagged in each container. The date of each selected ear was recorded as the day when the anthers of the florets of the central spikelet became visible.

### Temperature treatment and heat application

Two temperature regimes were applied. Plants of the control treatment (LT) were maintained from anthesis to maturity at 21/15°C (day/night temperature); the average daily air temperature was 19°C. Temperature treated (HT) plants were subjected to a temperature of 29/23°C (day/night) over the period of two to 13 daa. The average daily temperature was 27°C (S1 Fig). Each temperature regime was replicated twice (two enclosures for each treatment). In both regimes, the plants were watered *at libitum* to avoid drought especially when high temperatures were applied to the plants.

### Sampling procedures for morphological measurements and transcriptomic analysis

For each temperature treatment, sampling was conducted according to a thermal time calendar (°Cd). The °Cd are the sum of the average daily temperatures from anthesis to the day of harvest. For grain phenotyping and morphological measurements, grains were sampled i) at the beginning of the filling-phase (around 300°Cd, stage estimated from previous experiments (not shown)) and ii) at maturity. Only basal grains, grains #1 (G1) and # (G2) on the central spikelet of the ear, were sampled. At both stages (∼300°Cd and maturity), G1 and G2 grains were fresh weighed and their volume was determined using a water displacement method (pycnometry). Dorsal and lateral images of the grains were acquired under a stereomicroscope in order to measure the length, width and thickness of each grain at a later date. Then, for the grains sampled at ∼300°Cd, G1 were oven-dried at 65°C for 48h, then weighed to give the dry mass (DM). After sampling, G2 were immediately dissected and the endosperms isolated. The endosperms were then fixed at room temperature in a 1:3 (v/v) acetic acid-alcohol mixture. The endosperm cell number was determined by the method of Rijven and Wardlaw [37] as modified by Singh and Jenner [38] and the maximal endosperm cell number was recorded. At maturity, after fresh weight and microphotographs, G1 and G2 were oven-dried at 65°C for 48h and then weighed to give the dry mass. Ten replicates for each temperature treatment were made.

For transcriptomic analyses, ears were harvested at nine developmental stages: 0, 80, 120, 160, 180, 220, 240, 300 and 400°Cd and immediately frozen in liquid nitrogen to minimize the effects of wounding. All the grains within the central spikelet of the ear were sampled. For each developmental stage and for each treatment, two biological replicates were used and grains from 5–6 plants were pooled for each replicate.

### Statistical analysis of morphological measurements

For each variable, means were compared using a Student-Newman-Keuls (SNK) test at a 5% level of significance. The growth curve of DM accumulation per grain as a function of time expressed in days after anthesis was fitted to a logistic-3 parameters function, estimating the duration of the lag-phase and the filling-phase as well as the rates of DM accumulation for grains in each temperature treatment. The fitted equations were compared simultaneously between treatments by parallel curve analysis [39]. The significance of the comparison between treatments was based on Akaike’s Information Criterion (AIC; [40]). All statistical analyses were conducted using the SAS software (SAS Institute, 2002).

### Transcriptomic analysis

#### Total RNA extraction

Total RNAs were extracted from the grain sample pools using the method described by Capron *et al*. [41]. The RNAs was then treated with DNaseI (Invitrogen). RNAs integrity was checked using the RNA 6000 Nano assay bioanalyzer (Agilent).

#### Microarray design and analysis

Gene expression profiles were generated using NimbleGen microarrays for wheat (ref. A-MEXP-1928) with two biological replicates for each developmental stage in response to low and high temperature. Each microarray used comprised 40,656 UniGenes (Wheat UniGene set v55) with three different probes per UniGene. Two sets of dye-swapped experiments were performed to obtain a total of four replicate hybridizations per developmental stage. cDNA was synthesized from total RNA using the method described by Capron *et al*. [41]. Hybridizations were performed according to Capron *et al*. [41]. The raw data files generated from all chips were imported to Nimblescan 2.5 software for further analysis. In order to stabilize variations in data from different chips, the raw data were normalized using the GC-RMA (Gene Chip Robust Multi Array Analysis) algorithm. The microarray results were filtered to select UniGenes differentially expressed using LIMMA [42] with a *P-value*< 0.01. All the data have been submitted in MIAME-compliant form with the GEO accession number GPL17441.

#### Validation of wheat microarray data using qRT-PCR

To assess the reliability of microarray data, quantitative reverse transcription PCR (qRT-PCR) was used to measure the expression level of some selected genes according to Capron *et al*. [41] (S2 Fig). The Pearson correlation coefficient for each gene was calculated between the microarray and qRT-PCR data. The relative expression levels, determined by qRT-PCR, revealed excellent concordance with those determined using the arrays, with correlation coefficients ranging from 0.94 to 0.99. Given that the same variations in expression profiles were observed with both qRT-PCR and microarray analyses, the microarray results were deemed to be reliable.

#### Weighted gene coexpression network analysis (WGCNA)

The R package WGCNA was used for network constructions [43]. Networks were inferred separately for the low and the high temperature sets using the same parameters: A soft thresholding power of 12 (S3 and S4 Figs), networkType = "signed", minModuleSize = 60, maxBlockSize = 2000, corType = "pearson", deepSplit = 2. Unassigned genes were grouped in the “grey” module. Initial modules, whose expression profiles were very similar (eigengene correlation ≥ 0.75), were merged. For each co-expression module, the genes were annotated and significant over-represented biological processes and molecular functions were determined (see below).

#### Module comparison and network preservation between the LT and HT sets

Two methods were used in parallel to compare the networks in the LT and HT data sets. The ‘overlapTable’ function implemented in WGCNA was used to calculate overlap counts and Fisher’s exact test *p-values* for the low temperature (LT) and high temperature (HT) sets of module assignments. The WGCNA function ‘networkPreservation’ was used to evaluate the network module preservation between the low temperature and high temperature sets. These statistics quantify how density and connectivity profiles of modules defined in one data set (reference set) are preserved in a second data set (test set). This strategy was applied to measure reciprocal module preservation in the low temperature and the high temperature data sets (*i*.*e*. LT as reference data set and HT as test data set then *vice-versa*). The Preservation Zsummary statistic is given as a function of the module size and it was based on 200 permutation tests that took into account the preservation of both connectivity and density in a module. Zsummary<2 implies no evidence for module preservation, 2<Zsummary<10 weak to moderate evidence, and Zsummary>10 indicates a strong evidence for module preservation (Langfelder *et al*., 2011 [44]).

#### Identification of modules of co-expressed genes related to agronomic traits

The association between modules and agronomic traits (phenotypes) were estimated using the correlation between the phenotype and the module eigengene which summarizes the expression level of that module. This approach allowed for the identification of modules highly correlated to a given phenotype (Module Significance MS). In addition, Module Membership (MM) was measured using the correlation between the expression levels of a gene and the module eigengene. This measure reflects the connectivity of a gene with other genes in the module and was used to define the key or hub genes. Finally, we identified genes that have a high significance for agronomic traits in interesting modules using the gene significance (GS) measure [43]. The traits used for association analysis are summarized in S1 Table.

#### Gene annotation and gene enrichment analysis

We annotated the wheat unigenes (release #55) using Blast2GO [45]. Sequences were blasted with an e-value threshold of 10e^-20^ against the "green plants" database (NCBI). To identify significant enrichment in GO terms, the affymetrix ID was retrieved for each unigen and the Fisher’s exact test was performed for each module, using the whole wheat genome as background and a *p-value* threshold of 0.01 (Hochberg (FDR) as multi-test adjustment method) at AgriGo Server (http://bioinfo.cau.edu.cn/agriGO/).

## Results

Both ecophysiological and molecular analyses were carried out in parallel using SxB139 and SxB49 genotypes. While the two genotypes differed in terms of numerical results (for example the number of differentially expressed genes), they behaved in such a similar way that the same conclusions and comments could be drawn. Therefore, for clarity purposes, all the results will be presented on the genotype SxB139. The main results for the genotype SxB49 will be given in different sections as supplemental data.

### Seed morphological components were decreased by high post-anthesis temperatures

At the end of experiment, for each temperature treatment all the remaining mature spikes (50 spikes for control temperature and 70 spikes for high temperature) were sampled and grains were collected and weighed in order to estimate the Thousand Kernel Weight (TKW). TKW was reduced by 13.7% by high temperature.

**Fig 1:**
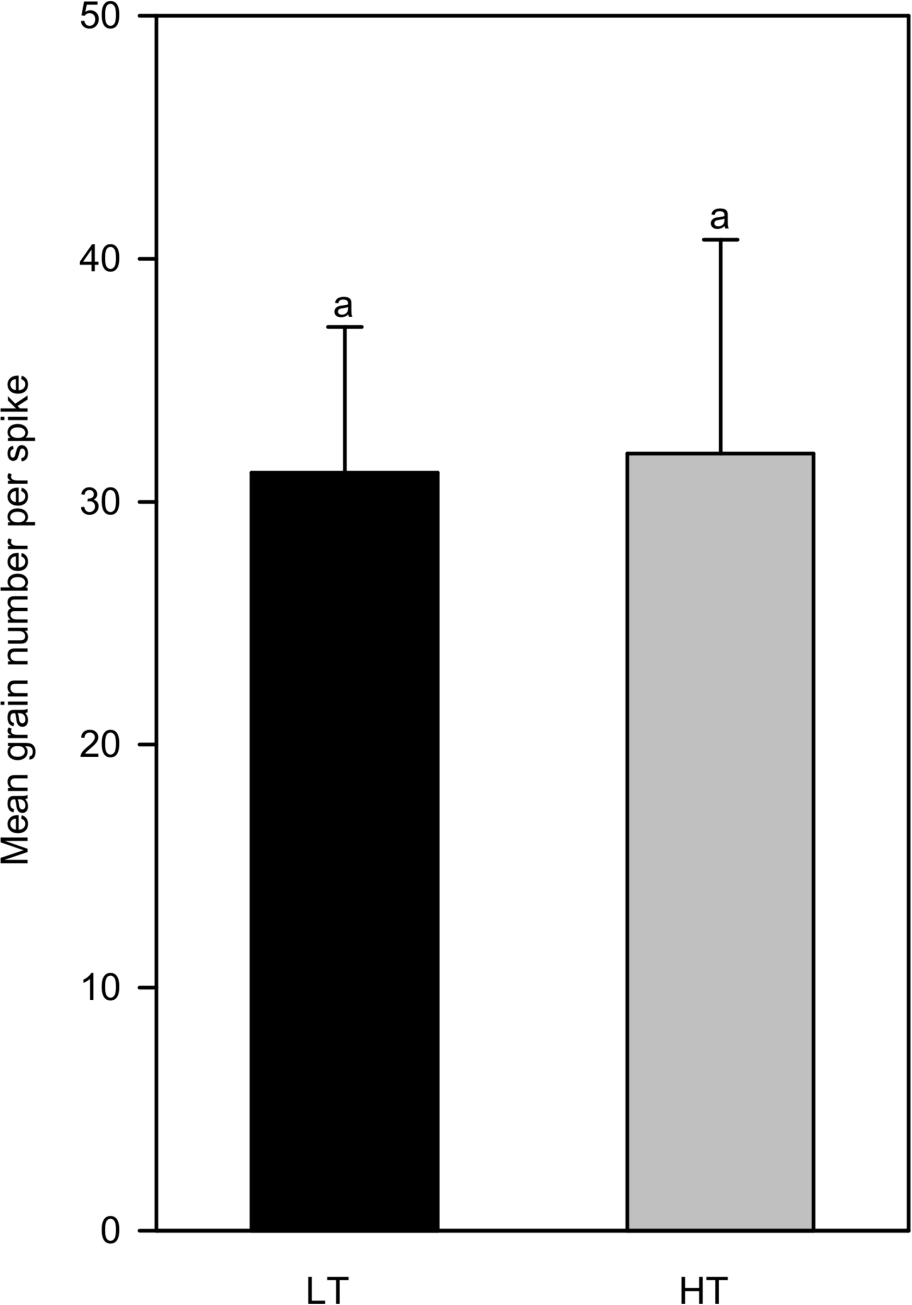
Mean number of grains per spike for control and heat treatment. The different letters above the bars indicate a significant effect of high temperature at a 5% level.

**Fig 2:**
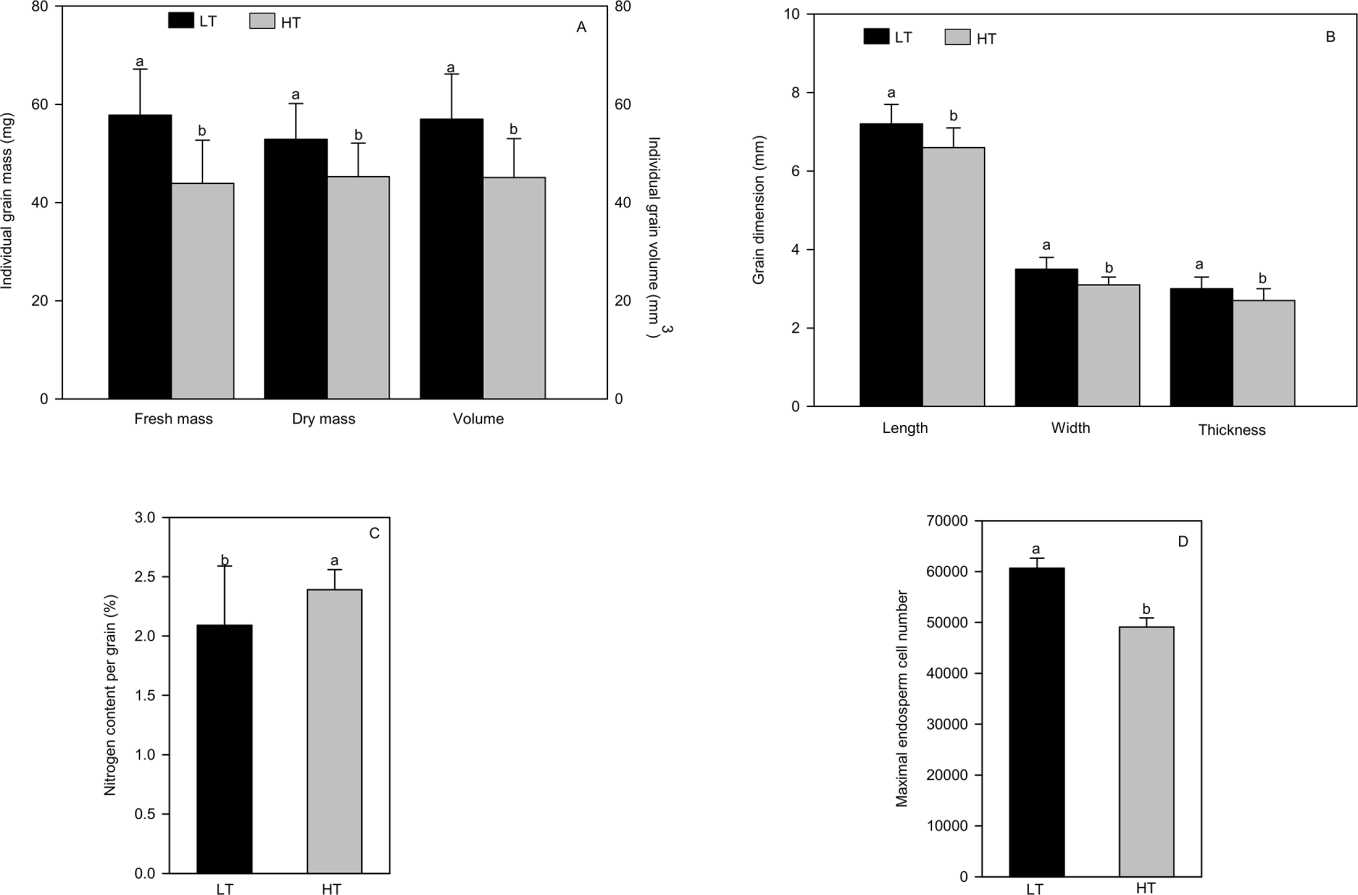
Effect of high temperatures on individual grain masses (fresh and dry) and volume (Fig 2A), and on grain dimensions (Fig 2B) for the genotype SxB139. High temperatures increased the nitrogen content of the grains significantly (Fig 2C) but decreased the maximal number of cells in the grain endosperm (Fig 2D). Means (n = 10), differing at a 5% level, are indicated by the different letters above the vertical bars.

In order to verify that the high temperatures under our experimental conditions had an impact on grain development, grains were phenotyped at maturity for morphological traits as a first step. As expected, the mean number of grains per spike was not affected by high temperature (Fig 1). In fact, high temperatures were applied on 2 daa in order to limit the effect of high temperatures on grain abortion. This experimental strategy was implemented to avoid a possible compensation of a lower number of grains per spike by higher masses of grains when subjected to high temperatures that could partly hide the real effect of heat. At maturity, all the masses and dimensions of individual grains (Fig 2A and 2B) (panels A and B in S5 Fig) were significantly decreased by high temperatures. For example, individual grain fresh mass was reduced by 24.0% in treated plants compared to control (LT) plants and grain dry mass (DM) was reduced by 14.4% at the same time. As for grain dimensions, length, width and thickness were decreased by 8.3, 11.4 and 10%, respectively, in heated plants compared to controls (LT). As grain C mass was reduced in the heated grains compared to the controls and as grain N mass was not significantly reduced by high temperatures, the grain nitrogen content per grain (expressed as a % of DM grain) was significantly enhanced by high temperature (Fig 2C) (panel C in S5 Fig). Finally, the final reduced size of the heated grains could be partly explained by a significant reduction of 19.1% in maximum endosperm cell number estimated at the beginning of the filling-phase of grain development (19 daa (285°Cd) and 11 daa (297°Cd) for LT and HT treatments respectively) (Fig 2D) (panel D in S5 Fig).

**Fig 3:**
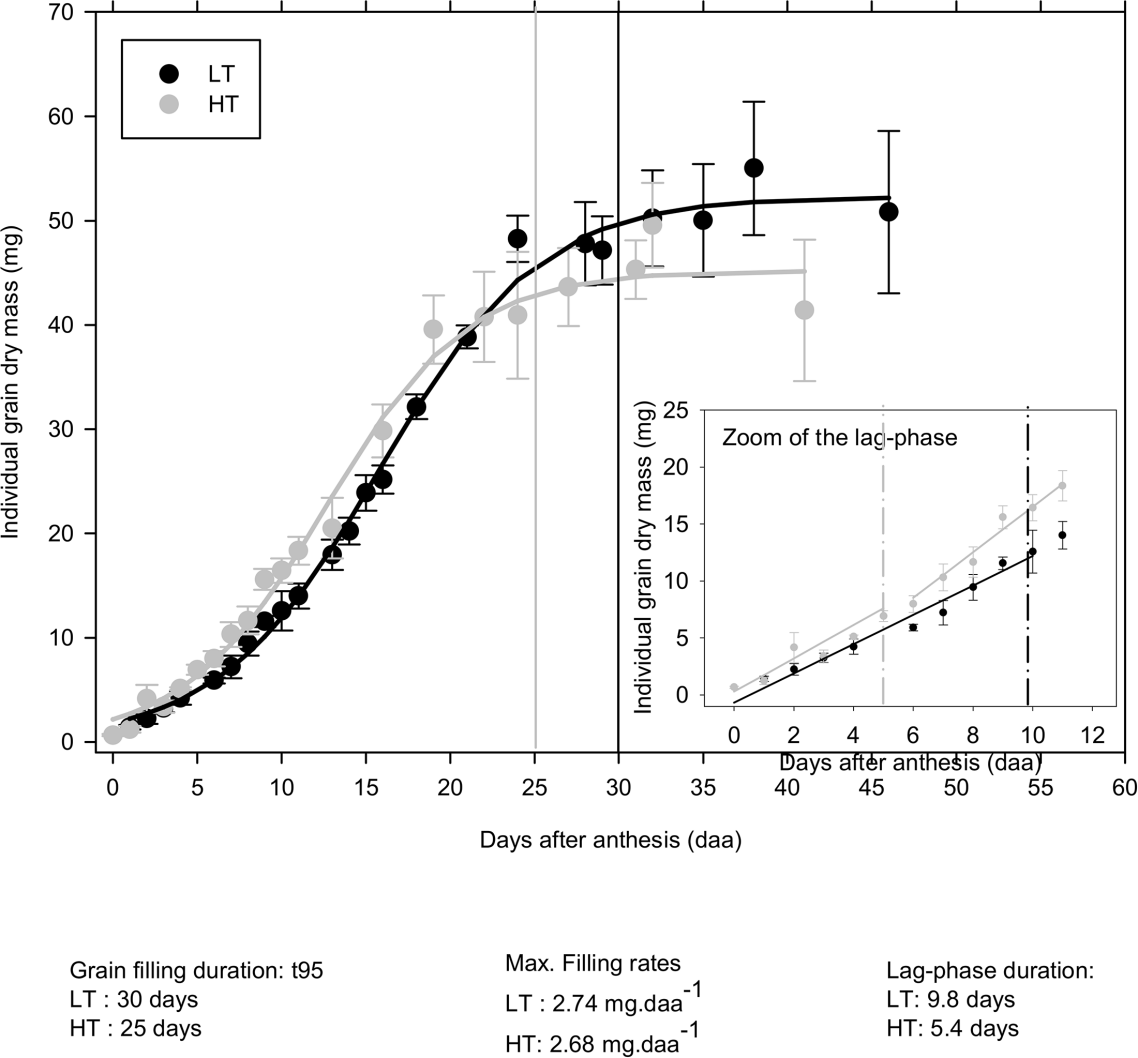
Effect of high temperatures on grain DM accumulation throughout grain development. For each temperature treatment, observed values were fitted to a logistic function. A zoom of the lag-phase has been inserted into the main graph. For each treatment, the vertical bars indicate the end of the lag- or filling-phases.

To test if the differences in final grain mass due to high temperatures are reflected in the timing and rate of grain filling, the individual grain DM was measured everyday throughout grain development during the lag-phase and every two days during the filling-phase. For this purpose, the duration of the lag- and filling-phases were expressed in days, as the final grain DM mainly depends on the number of days of source functioning (C assimilation by leaves). Comparison of fitted functions between heat treatments showed a statistical effect of high temperatures on grain DM accumulation. Duration of both lag- and filling phases was reduced by 45% and 16.7% respectively for heated plants compared to the control ones (Fig 3 and insert). As expected, the DM filling rate into the grain was not significantly different between heat treatments (estimated DM filling rate: 2.74 mgDM.daa^-1^ for the control (LT) grains and 2.68 mgDM.daa^-1^ for the heat treatment (HT) ones).

### Hierarchical clustering

**Fig 4:**
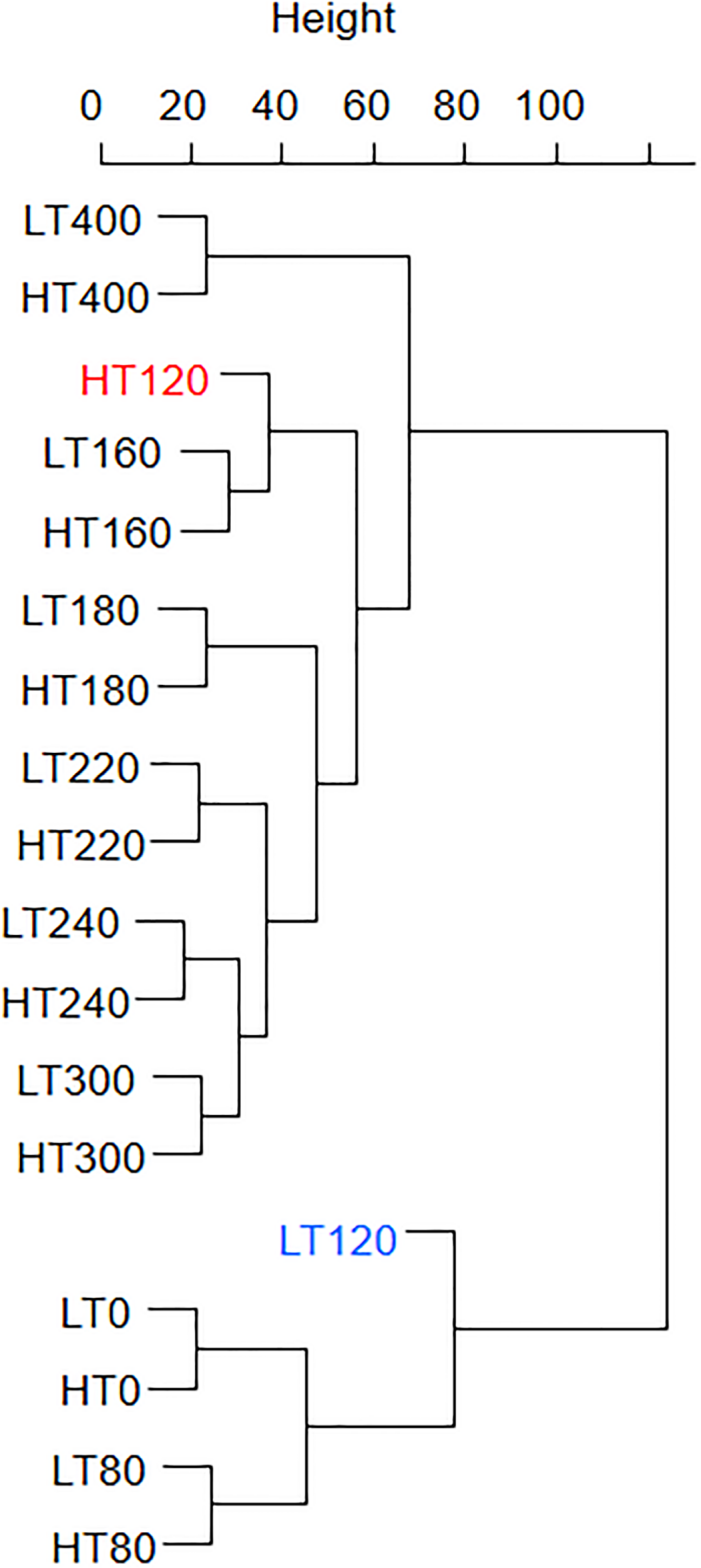
Wheat grain samples clustering based on euclidean distance and complete linkage method. The median expression of 6,258 DEGs were retrieved in the nine developmental stages (denoted S0 trough S400 in °Cd) and the two growth conditions (LT, 19°C and HT, 27°C) and then used for hierarchical clustering of the samples.

Reprogrammation of the expression of the entire wheat genome was first assessed by using hierarchical clustering of the whole set of wheat unigenes using euclidean distance. As shown in Fig 4, developmental stages grouped together independently of the temperature regime, except the 120°Cd stages which displayed the greater difference between low and high temperature. The 120°Cd HT clustered with 160°Cd stages. These observations suggest that significant transcriptional changes occur in response to warming during the transition that encompasses the 120°Cd stage.

### Differential expression analysis

It was expected that a high temperature would induce changes in the expression profile when compared to a low temperature. Limma analysis (time-course procedure) uncovered two types of differentially expressed genes (DEGs): a large set contained more than 12,000 DEGs and corresponded to the genes whose expression varied according to the developmental stage and independently of the temperature regime. These genes were then called development-related genes. A second set contained 6,258 DEGs whose expression was affected by temperature at one or more of the developmental stages.

**Fig 5:**
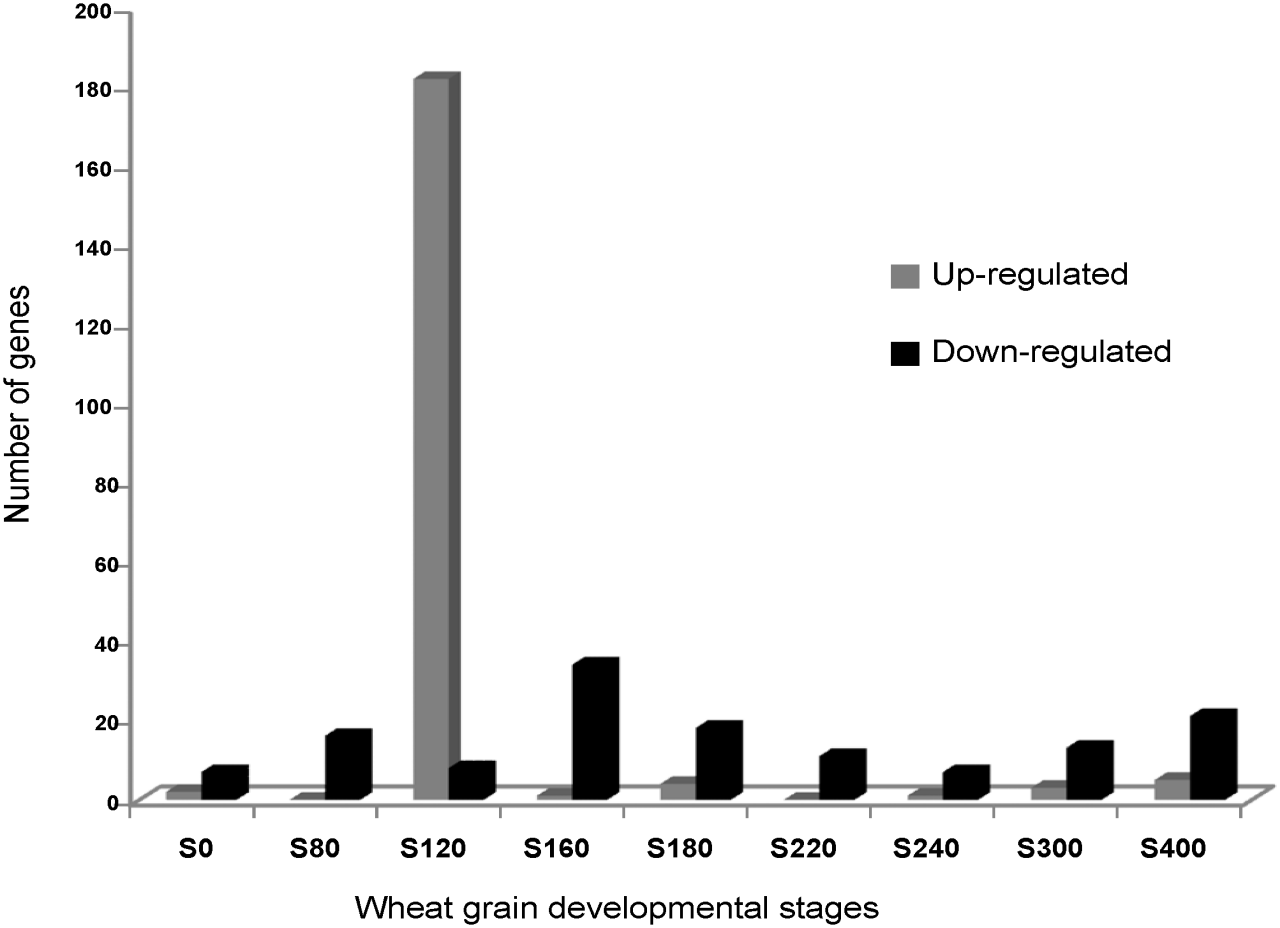
Number of up-regulated (log2FC ≥ 1.5) or down-regulated genes (log2FC ≤ -1) was plotted for each developmental stage in wheat grain.

To gain insight into the effect of temperature on the expression of the genes, the DEGs were filtered out on the basis of their log2 Ratio between high (HT) and low temperature (LT). The genes whose log2Ratio was ≥ 1.5 were considered as up-regulated, and those whose log2Ratio was ≤ -1 were considered as down-regulated. Using these criteria, 196 genes were up-regulated and 117 were down-regulated. While the down-regulated genes were evenly distributed over developmental stages, 182 out of the 196 up-regulated genes were detected in the 120°Cd stage (Fig 5 and S2 Table). Concerning the SxB49 genotype, 212 genes were up-regulated and 480 were down-regulated. Consistent with the hierarchical clustering results (Fig 4), this finding indicates that this was the stage where the most differences were observed.

Both the up- and down-regulated genes were then annotated and their enrichment in particular metabolic pathways was investigated using a Fisher’s exact test and the whole wheat genome as background. While no significant enrichment was observed in the down-regulated set, the up-regulated genes were found enriched in genes involved in “pathogenesis” (GO:0009405, *pValue* = 7e^-10^, FDR = 3.50e^-09^) and highly enriched in “nutrient reservoir activity” (GO:0045735, *pValue* = 7.3e-^51^, FDR = 7.3e-^51^). In particular, this set contained 141 unigenes coding for seed storage proteins. For instance, it included alpha-gliadin (63 unigenes *i*.*e*. Ta#S13005380, log2FC = 3.9), high or low molecular weight glutenin subunits (62 unigenes *i*.*e*. Ta#S46915591, log2FC = 3.1). In addition, the set contained one gene coding for ADP-Glucose Pyrophosphorylase small subunit or AGPase (Ta#S16227636, log2FC = 1.97605 at 120°Cd). AGPase synthesizes ADP-Glucose which serves as glucose donor for starch synthesis within the plastid (S2 Table).

### Genes co-expression analysis using WGCNA

#### Construction of LT and HT co-expression networks

Weighted gene coexpression network analysis WGCNA [46] was used to generate two separate networks (modules) of highly correlated DEG genes in LT and HT experiments. As shown in S3 Table, 32 modules were identified in the LT network. The smallest module contained 86 genes (steelblue module) and the largest one contained 687 genes (turquoise module). In the HT network, 30 modules were identified and there were two small modules with 90 and 91 genes (saddlebrown and skyblue modules). The largest module contained 670 genes (turquoise module).

#### Modules preservation between LT and HT co-expression networks

As the LT and HT networks were inferred separately, it was important to assess the conservation between the modules. In a first approach, a Fisher’s exact test was used to monitor the overlapping in gene content between the modules in LT and HT conditions (see Materials and methods). Several modules shared a high number of genes, in particular, the LT-turquoise and HT-turquoise shared 440 genes (p-value = 6.47e^-316^) (S6 Fig). In a second approach, the ‘networkPreservation’ function was used to evaluate the network module preservation between the LT and HT sets. These statistics quantify how density and connectivity profiles of modules defined in one data set (reference set) are preserved in a second data set (test set). As shown in S7 Fig, both Preservation Median Rank and Preservation Zsummary statistics indicate that both connectivity and density are strongly preserved (Zsummary > 10). In particular, there was strong evidence of preservation in turquoise modules (Zsummary ≥ 50).

#### Identification of modules related to agronomic traits

In addition to the detection of modules of genes with highly correlated expression profiles, WGCNA allows the measure of correlation between modules and traits. For this purpose, the “Module Eigengene” (ME), which summarizes the expression profile of a given module, was used to search for a correlation with traits. Three modules were found highly correlated with traits in the LT set. Tan module was negatively correlated with dry matter (cor = -0.91, p = 6e^-04^) and fresh weight (cor = -0.81, p = 0.008). Pink module was negatively correlated with several traits, cell number (cor = -0.99, p = 1e^-06^), thickness (cor = -0.92, p = 4e^-04^) and length (cor = -0.95, p = 1e^-04^). Turquoise module was positively correlated with length (cor = 0.92, p = 5e^-04^) and cell number (cor = 0.98, p = 2e^-06^) (S8 Fig). Likewise, several modules were found highly correlated with traits in the HT set. Purple module was negatively correlated with fresh weight (cor = −0.90, p = 0.001), volume (cor = −0.93, p = 2e^−04^), length (cor = −0.96, p = 3e^-05^), width (cor = −0.95, p = 7e^−05^), thickness (co r = −0.96, p = 3e^−05^). Cyan module was positively correlated with dry matter (cor = 0.94, p = 1e^−04^). Turquoise module was positively correlated with volume (cor = 0.81, p = 0.008), length (cor = 0.90, p = 0.001) and cell number (cor = 0.97, p = 2e^−05^) (S9 Fig).

**Fig 6:**
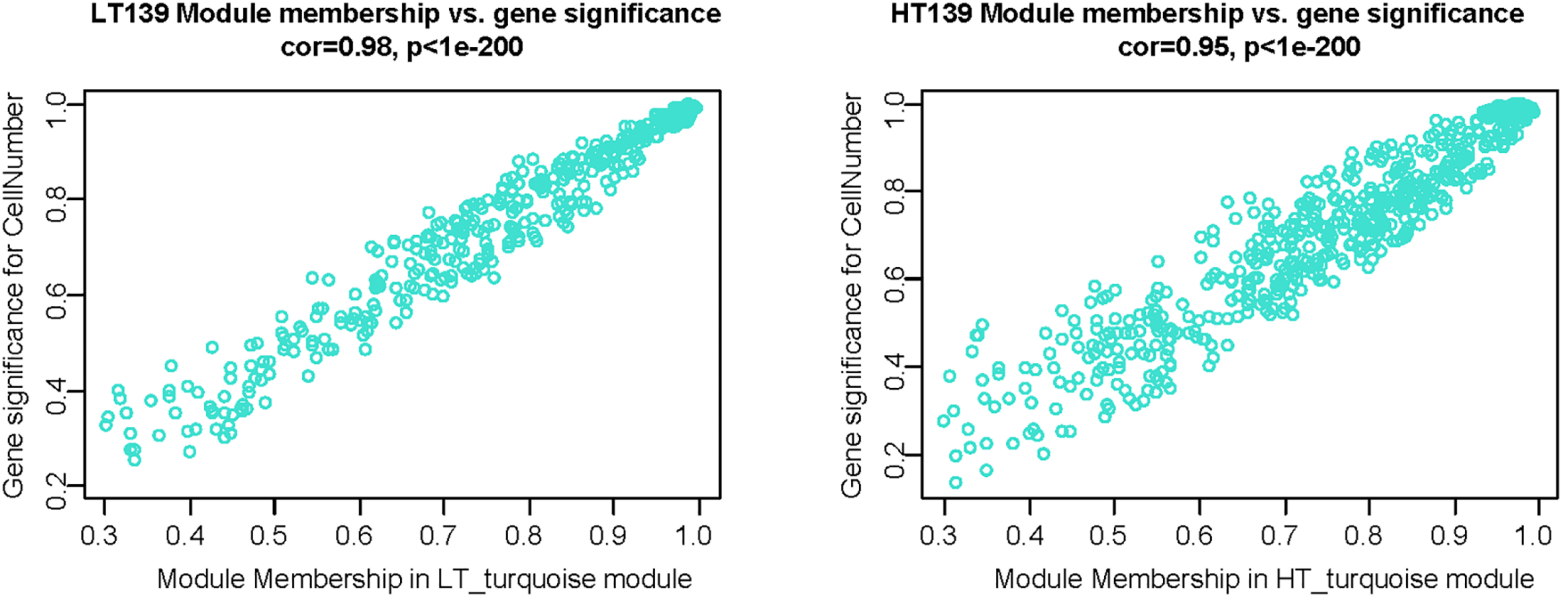
A scatterplot of Gene Significance (GS) for cell numbers in the endosperm vs Module Membership (MM) in the turquoise modules in LT (19°C, left panel) and HT (27°C, right panel) networks. There is a highly significant correlation between GS and MM in these modules.

As turquoise modules were both highly correlated with cell number traits, the association of individual genes within these modules with this trait was further investigated. For this purpose, Gene Significance (GS) and Module Membership (MM) were quantified (see Materials and methods). Fig 6 shows the relationship between the GS and MM in the LT and HT turquoise modules (cor = 0.98, p<1e^-200^, and cor = 0.95, p<1e^-200^ respectively). This indicates that hub genes of the turquoise modules are likely to be highly correlated with cell numbers in the endosperm.

### Effect of warming on the expression of genes related to agronomic traits

**Fig 7:**
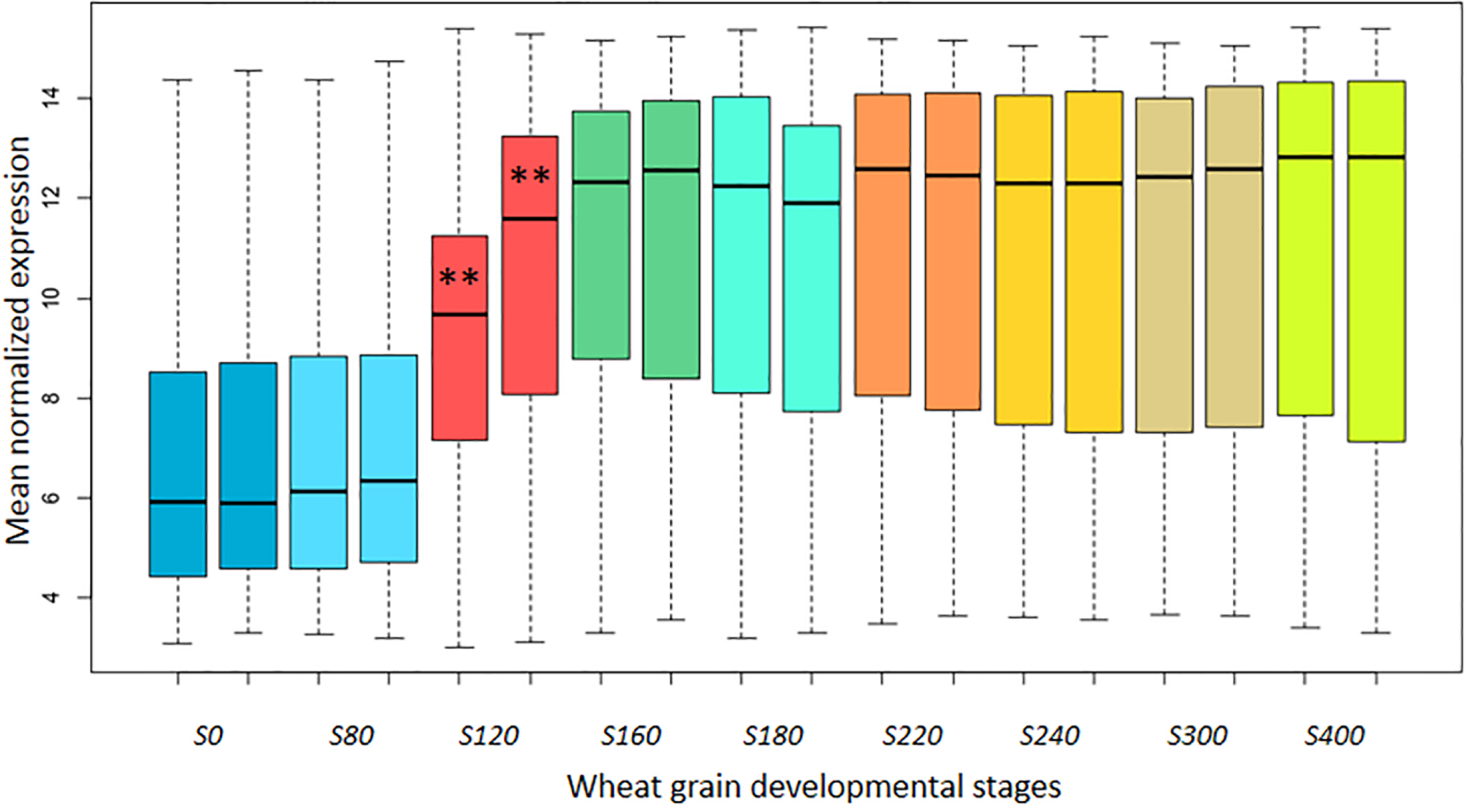
Mean expression of top 200 genes in turquoise modules associated with endosperm cell numbers, across nine grain developmental stages at 19°C (LT experiment) or 27°C (HT experiment) for SxB139. The mean normalized expressions of top 200 genes displaying the greatest Gene Significance of the association with the trait "Cell number in the endosperm" were retrieved and plotted according to the wheat grain developmental stages. At each time-point, the mean expression of the genes at 19°C (LT experiment) and at 27°C (HT experiment) were plotted alongside with the standard deviation. Asterisks denote highly significant differences (TukeyHSD test, p<0.001).

As the turquoise modules in the LT and HT networks were highly correlated with several agronomic traits and, in particular, with the number of cells in the endosperm, we further considered the expression profile of these modules. Therefore, the top 200 genes showing the highest Gene Significance score (*i*.*e*. high correlation with the trait) were retrieved (S4 Table) and their mean expression profiles were compared in low and high temperature regimes. These sets of top 200 genes contained all the genes coding for seed storage proteins and some starch-related enzymes. As indicated in Fig 7, both sets behaved similarly in low and high temperatures (S10 Fig). The mean expression was low at 0°Cd and increased steadily to reach a plateau which lasted throughout the rest of the developmental stages. However, while the maximum of the expression (plateau) was reached at 160°Cd under low temperature, this plateau commenced earlier (at the 120°Cd stage) under high temperature. It is noteworthy that the shapes of the profiles were almost identical and that the plateau phases could be superimposed. Consistent with the results of clustering, the shift of the profiles was significant at the 120°Cd stage which may indicate that this stage is highly responsive to a warming applied at anthesis and lasting up to 250°Cd.

### Gene enrichment in WGCNA modules

The modules in the LT and HT networks were annotated then searched for enrichment in specific pathways. Only two out of 32 modules in the LT network were found to be significantly enriched in particular biological processes or cellular functions (p<0.01). The royalblue module was enriched in defense response (GO:0006952) and response to stress (GO:0006950) with FDR = 0.0032 and FDR = 0.0035 respectively. The turquoise module was enriched in nutrient reservoir activity (GO:0045735), FDR = 3.4e^-34^ and endopeptidase inhibitor activity (GO:0004866), FDR = 4.5e^-09^. Interestingly, out of 30 modules detected in the HT set, only the turquoise module, was found to be significantly enriched in nutrient reservoir activity (GO:0045735), FDR = 1.90e^-32^ and endopeptidase inhibitor activity (GO:0004866), FDR = 2.3e^-07^ (S4 Table).

A careful examination of the genes within the turquoise modules in the LT and HT experiments indicated that they contained 147 and 146 genes respectively annotated as coding for seed storage proteins, among which, 146 were common such as gamma-gliadin (Ta#S12902257), low molecular weight glutenin (Ta#S24517516) and high molecular weight glutenin (Ta#S13074293). They also contained respectively 10 and 11 genes coding for proteins involved in grain quality such as grain softness protein (Ta#S32594136) and puroindoline (Ta#S50384070).

**Fig 8:**
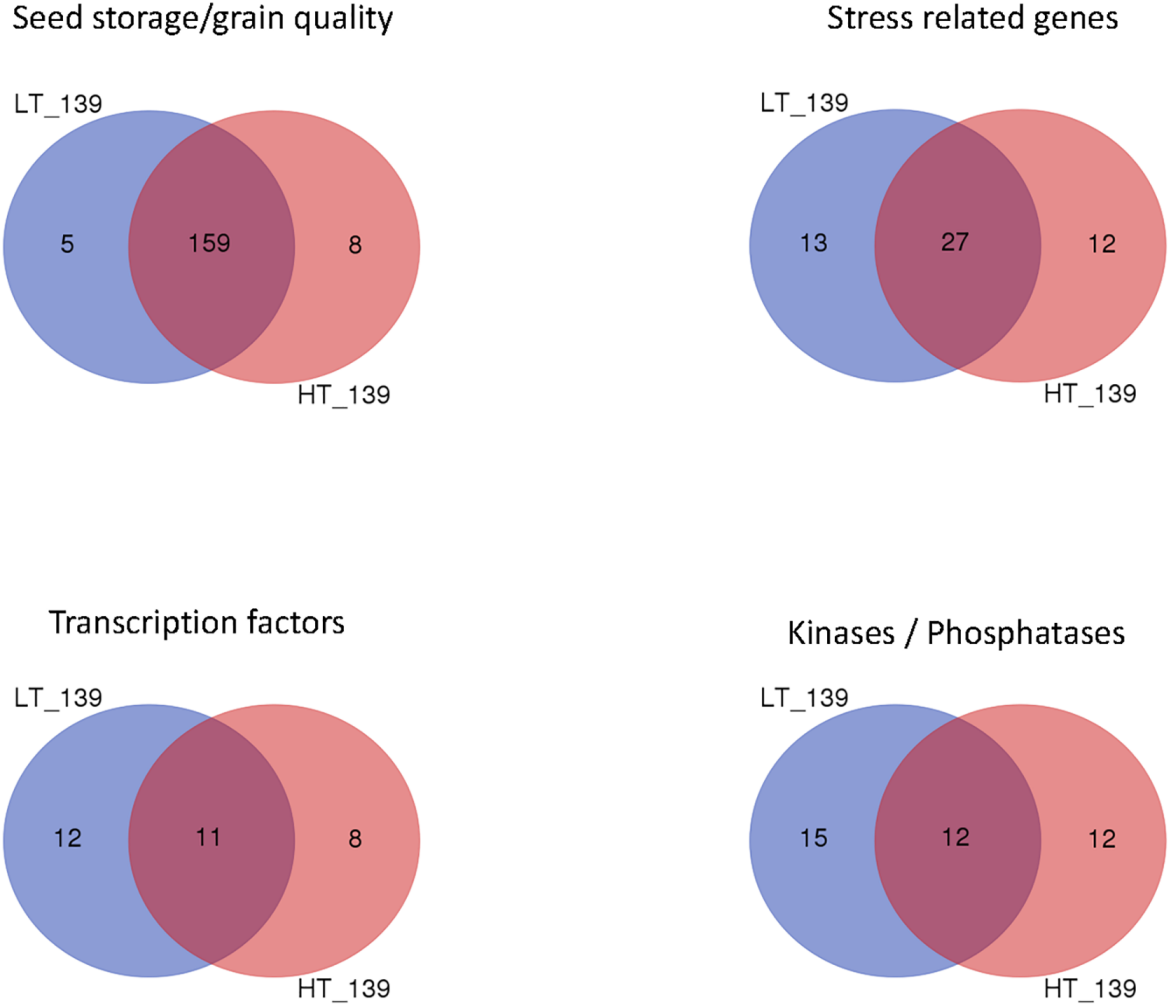
Gene contents of the turquoise modules associated with cell number trait in wheat grain. The genes were annotated and grouped according to their biological functions. Four biological functions with significant overlapping between the LT-turquoise and HT-turquoise modules were detected. Numbers indicate the number of genes in each class.

In addition, the two modules shared genes related to stress response in wheat grain such as alpha-amylase inhibitor (Ta#S13260187) and heat shock protein 90 (Ta#S17888158) (S5 Table and Fig 8). Interestingly, while a few genes related to starch metabolism were found, two unigenes coding for ADP-glucose pyrophosphorylase (AGPase) were shared between the two modules (Ta#S16227636 and Ta#S12922948). This enzyme catalyzes the conversion of Glucose-1-P and ATP to ADP-Glucose and pyrophosphate which represents a rate-limiting step in starch synthesis in maize [47].

## Discussion

### Effect of warming on grain yield traits (negative impact on yield components)

The effects of temperature have long been the subject of numerous publications on different plant species. However, most of these studies have focused on analyzing the effects of extreme temperatures that can be sub-physiological or non-physiological, with dramatic consequences in plants. Conversely, the effects of moderate elevation (warming) in a range of physiological temperatures could have less visible phenotype modifications depending on the stage of application, the duration and the intensity of this treatment. In bread wheat, Wan *et al*. [32] showed that an increase in temperature up to 28°C accelerated grain development during the first 7 days after anthesis. Consequently, the control (19°C) had progressed by the equivalent of 12 days, meanwhile, the treated (27°C) progressed by the equivalent of 17 days, giving a net acceleration of about 5 days under these conditions.

In this work, warming reduced the TKW and the dry mass of individual grains by respectively 13.7% and 14.4%, and this reduction was not due to a decrease of the filling rate. We found that the maximum filling rate (2.74 vs 2.68 mg dry matter per daa) is not affected by temperature. In contrast, the filling duration was significantly reduced from 30 days at 19°C to only 25 days to reach 95% of the maximum nutrients accumulation at 27°C.

Although the stage and the duration of application of a high temperature are not identical, this result coincides with the observations of Wan *et al*. [32] in wheat, as well as with those of Boden *et al*. [48] in *Brachypodium distachyon*. Similarly, our results are consistent with those found by Ferris *et al*. [14] who showed that 10°C above ambient temperature negatively affected all the agronomic traits measured in wheat.

### Effect of warming on the expression of the genes in wheat grain

Globally, the high temperature treatment applied in our experiment (19°C vs 27°C) seems to induce few extreme variations in the expression of genes during wheat grain development. Indeed, the absolute number of differentially expressed genes linked to temperature is relatively low compared to the number of genes linked to development.

As a matter of fact, a few genes exhibited a high expression variation (Fold Change ≥ 2) upon warming. However, it is worthy to note that those genes are different from the genes generally over-expressed under "severe heat stress" conditions commonly reported in the literature. In particular, significant induction of HSP genes and HSF transcription factors is a hallmark of a response to a severe heat stress. For instance, when seeds of oilseed rape (*Brassica napus*) were subjected to a temperature of 35°C for five hours, several heat stress marker genes were strongly induced. This set included 13 HSFs and 91 HSPs, which were strikingly up-regulated by an average of 36.5-fold [49]. Indeed, in our study, 26 genes were annotated as "Heat stress genes" such as HSP70 or HSP90 (S2 Table), however, only one gene (Ta#S3268284) coding for a "23.2 kDa heat shock protein-like" was found up-regulated with a log2FC = 2.1 in stage 240°Cd. A similar observation was reported in wheat by Wan *et al*. [32], who even found that a gene coding for an HSP70 was down-regulated by warming.

Therefore, it is arguable that the low intensity of the "stress" applied in our study induces only a moderate reaction as observed in Arabidopsis *in vitro* [50]. Although heat stress was not directly addressed in their study, Claeys *et al*. [50] analyzed several other abiotic stresses and found that plant growth and gene expression responses to stress are highly dose-responsive, suggesting the existence of a very sensitive machinery which is fine-tuning the molecular responses in accordance with the stress level.

### Co-expression analysis and the identification of trait-related modules

WGCNA is one of the numerous methods used for the inference of gene networks from transcriptomic data [43]. In addition, this approach could be used to uncover correlations between quantitative traits and the expression profiles of genes. In this study, a set of differentially expressed genes between low and high temperature assays were identified and retained for the construction of the co-expression gene networks. As several agronomic traits were measured in the two experiments, two networks have been independently inferred in both assays, yielding 32 and 30 modules in LT and HT respectively. A significant correlation of agronomic traits was found between several modules. In particular, 2 modules (turquoise modules) were found correlated to cell numbers in the endosperm and grain length in both temperature treatments. Annotation of the genes within these modules showed a significant gene enrichment (Fisher’s exact test) in nutrient reservoir activity. In addition, they are of similar size (687 and 670 genes respectively) among which, 440 are common, indicating a high degree of conservation. Thus, the two modules are deemed functionally equivalent. The significant effect of the high temperature on the mean expression of the genes in these modules is an acceleration at the 120°Cd stage, a plateau is attained at this stage in the high temperature assay, while such a plateau is reached with a significant delay at 160°Cd in the low temperature assay.

Collectively, these results indicate, that i) a set of genes involved in nutrient reservoir activity (in particular seed storage proteins and to a lesser extent sugar metabolism) is significantly associated with yield components in wheat grains (cell numbers), and ii) expression of genes involved in nutrient reservoir activity is hastened by a moderate high temperature. These results also suggest that the early acceleration of starch and seed storage proteins accumulation may somehow reduce the cell division duration, leading to the reduction of the final seed size. Our results are consistent with the finding of Sekhon *et al*. [51] who developed two divergent maize populations, differing in seed weight and seed size by magnitudes of 4.7-fold and 2.6-fold respectively. Phenotypic comparison of these populations indicated a significant reduction of the duration of the filling-phase in the small seed population. They also identified a critical stage (12 days after pollination DAP) where a substantial change in the expression of key genes involved in starch and seed storage protein synthesis was observed. For example, the major genes involved in starch biosynthesis such as sucrose synthase, a large subunit of ADP-Glc pyrophosphorylase, and starch branching enzyme1, had higher levels of expression in the endosperm of small seed inbreds at 12 DAP relative to large seed inbreds. Similarly, the genes encoding maize seed storage proteins (zein proteins) also had higher expression in small seed inbreds at 12 DAP. The authors suggested that carbohydrate status appears to be a key factor determining seed size by controlling the time of initiation and the rate and duration of endosperm cell division. In contrast, when barley caryopses were subjected to severe heat stress (42°C for up to six hours), Mangelsen *et al*. [29] observed a down-regulation of genes related to photosynthesis and starch synthesis leading to a net reduction in yield. One hypothesis explaining a significant correlation between the expression of genes involved in nutrient reservoir activity (sugar and seed storage proteins) and at least one yield component (*i*.*e*. cell numbers) is that the sugar and nitrogen act not only as nutrients but also as signals triggering adaptive developmental responses. In particular, emerging data indicate that sugar-derived signaling systems, including trehalose-6-phosphate (T6P), sucrose non-fermenting related kinase1 (SnRK), and the target of rapamycin (TOR) kinase complex play important roles in regulating plant development by modulating nutrient and energy signaling especially under abiotic stresses where sugar availability is low [52]. It has also been suggested that cell division could be inhibited due to a decreased glucose level under stress conditions leading to fruit and seed abortion at harvest [52]. Interestingly, nitrate and amino acids also activate TOR kinase by unknown mechanisms in Arabidopsis [53] suggesting that the plant TOR signaling network may also sense the nitrate and amino-acids status to control plant growth. Hence, in an attempt to depict a link between grain development, nitrogen/soluble sugar status and warming, a hypothetical model could be proposed in wheat. Under optimal temperatures, there is a balance between the growth of the different grain compartments and the accumulation of starch and storage proteins. Upon warming, there is a premature onset of transcription of genes related to starch and seed storage proteins biosynthesis. The early accumulation of storage compounds may divert the nitrogen and carbon resources towards nutrients accumulation, thus creating an imbalance in the partitioning of these resources at the expense of growth. In addition, the nitrogen and soluble sugar status may act as a signal to restrict cell divisions in the endosperm. The duration of the cell division phase is then shortened, eventually leading to smaller grains.

## Supporting information

S1 Fig Temperature treatment (panel A) and the timing (panel B) of their application according to grain development. Two temperature treatments were applied; mean day temperature: 19°C for low temperature condition (LT) vs 27°C for high temperature conditions (HT). HT were only applied during the lag-phase of grain development.
(PDF)

S2 Fig Validation of microarray data by comparing the expression estimates from NimbleGen wheat microarrays and quantitative reverse transcription PCR for some differentially expressed genes. Red graphs represent DNA microarray data depicting the expression intensity of each transcript (left y-axis). Blue graphs depict qRT-PCR results (right y-axis representing the relative expression level) during the nine developmental stages (x-axis representing the thermal time after anthesis (°Cdays)). The correlation coefficient (R) between the two graphs is indicated for each gene.
(PDF)

S3 Fig Analysis of network topology in the low temperature experiment (19°C) for various soft-thresholding powers. The left panel shows the scale-free fit index (y-axis) as a function of the soft-thresholding power (x-axis). The right panel displays the mean connectivity (degree, y-axis) as a function of the soft-thresholding power (x-axis).
(PDF)

S4 Fig Analysis of network topology in the high temperature experiment (27°C) for various soft-thresholding powers. The left panel shows the scale-free fit index (y-axis) as a function of the soft-thresholding power (x-axis). The right panel displays the mean connectivity (degree, y-axis) as a function of the soft-thresholding power (x-axis).
(PDF)

S5 Fig Effect of high temperatures on individual grain masses (fresh and dry) and volume (panel A), on grain dimensions (panel B), the nitrogen content of grains (panel C) and the maximal number of cells in the grain endosperm (panel D) for the genotype SxB49. Means (n = 10) differing at a 5% level are indicated by different letters above vertical bars.
(PDF)

S6 Fig Correspondence of the LT and HT modules. Each row of the table corresponds to one of the 32 modules in the LT network (19°C) and each column corresponds to one of the 30 modules in the HT network (27°C). Numbers indicate gene counts in the intersection of the corresponding modules and the -log(p), with p being the Fisher's exact test p-value for the overlap of the two modules.
(PDF)

S7 Fig Composite preservation statistics of the LT modules in the HT samples. A. The composite statistic medianRank (y-axis) as a function of the module size. Each point represents a module, labeled by color. Low numbers on the y-axis indicate a high preservation. B. The summary statistic Zsummary (y-axis) as a function of the module size. Each point represents a module, labeled by color. The dashed red and blue lines indicate the thresholds Z = 2 and Z = 10, respectively.
(PDF)

S8 Fig Relationships between the 32 modules eigengenes of co-expressed genes (rows) in grain wheat grown at low temperature (19°C) and agronomic traits (column). Correlation and p-value are given for each module-trait combination.
(PDF)

S9 Fig Relationships between the 30 modules eigengenes of co-expressed genes (rows) in grain wheat grown at high temperature (27°C) and agronomic traits (column). Correlation and *p-value* are given for each module-trait combination.
(PDF)

S10 Fig Mean expression of top 200 genes in turquoise modules associated with endosperm cell numbers, across nine grain developmental stages at 19°C (LT experiment) or 27°C (HT experiment) for SxB49. The mean normalized expressions of top 200 genes displaying the greatest Gene Significance of the association with the trait "Cell number in the endosperm" were retrieved and plotted according to wheat grain developmental stages. At each time-point, the mean expression of the genes at 19°C (LT experiment) and at 27°C (HT experiment) were plotted alongside with the standard deviation. Asterisks denote highly significant differences (TukeyHSD test, p<0.001).
(PDF)

S1 Table Agronomic traits measured during the nine developmental stages in wheat grain grown in low temperature (19°C) or in high temperature (27°C). For each stage and trait, values are estimated from the fitted equations of the growth curve of observed values as a function of time. In parentheses, the standard-error of the predicted mean value is indicated.
(XLSX)

S2 Table Median of normalized expression of 6258 DEGs at LT (19°C) and HT(27°C) in the nine developmental stages of wheat grain. LT0-400 and HT0-400 indicate developmental stages given in °Cd units.
(XLSX)

S3 Table Wheat DEGs were filtered using log2FC. Genes with log2FC ≥ 1.5 were considered as up-regulated and colored in red. Genes with log2FC ≤ -1 were considered as down-regulated and colored in green.
(XLSX)

S4 Table Annotation and distribution of the differentially expressed genes (DEGs) among the modules in the LT (19°C) and HT (27°C) networks. (XLSX)

S5 Table Annotation of the gene contents in turquoise modules in the LT (19°C) and HT (27°C) networks. Some metabolic pathways are color-coded (yellow: seed storage, green: grain quality, orange: stress-related, blue: Transcription factor, magenta: Kinase and Phosphatase, red: sugar metabolism). GS.CellNumber and p.GS.CellNum: Gene significance of the association with the trait "Cell number" and p-Value. MM.turquoise and p.MM.turquoise: Module membership and the p-value associated with.
(XLSX)

## Abbreviations

DAA: Days After Anthesis
WGCNA: Weighted Gene Coexpression Network Analysis
