# Supplementary figures and images for: Coexpression network and phenotypic analysis identify metabolic pathways associated with the effect of warming on grain yield components in wheat
Source: PLoS One. 2018 Jun 25;13(6):e0199434. doi: 10.1371/journal.pone.0199434 (PMC6016909; doi:10.1371/journal.pone.0199434)

S1 Fig

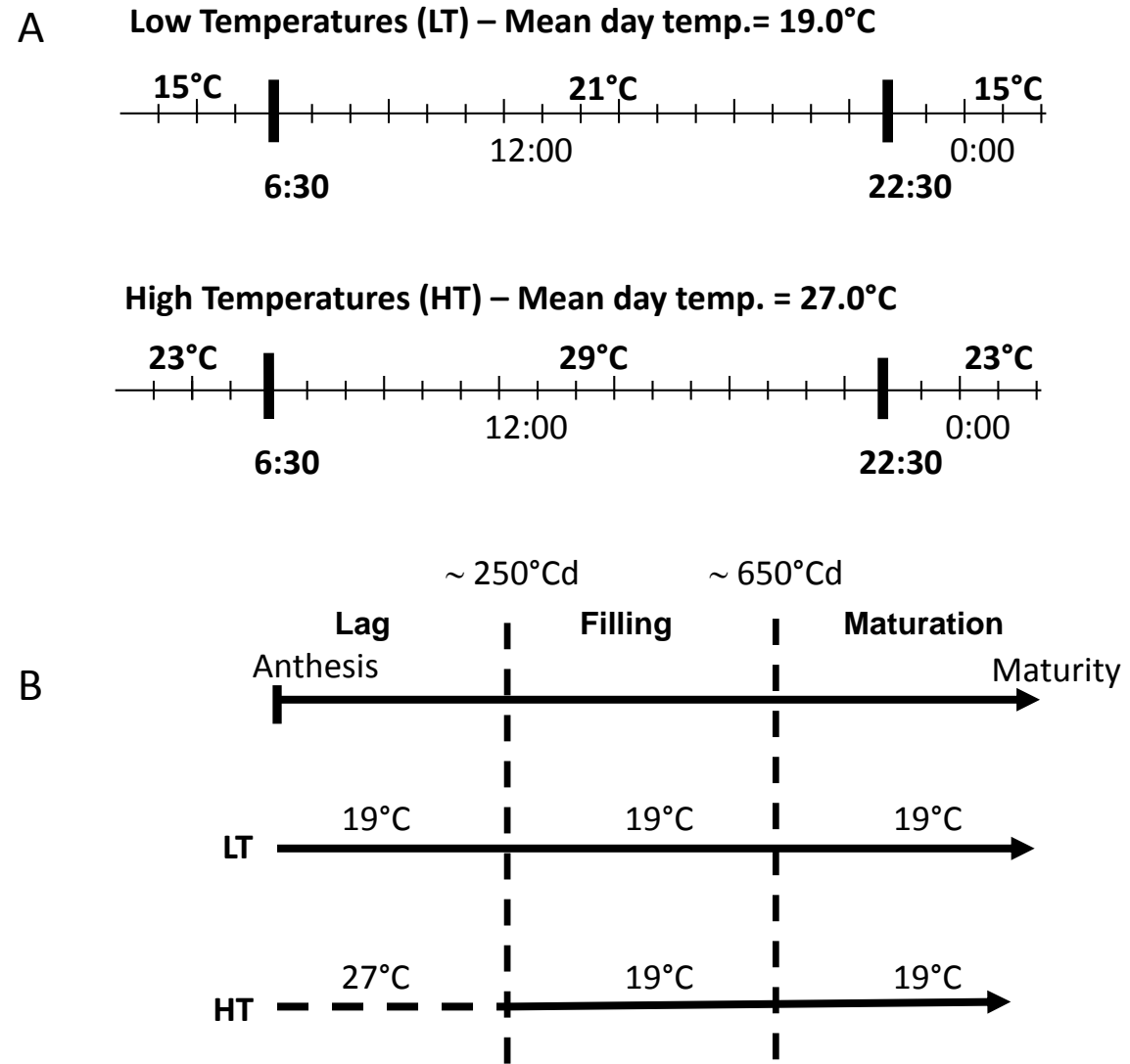

Supplement: S1 Fig — Two temperature treatments were applied; mean day temperature: 19°C for low temperature condition (LT) vs 27°C for high temperature conditions (HT). HT were only applied during the lag-phase of grain development. (PDF) [file pone.0199434.s001.pdf]

S2 Fig

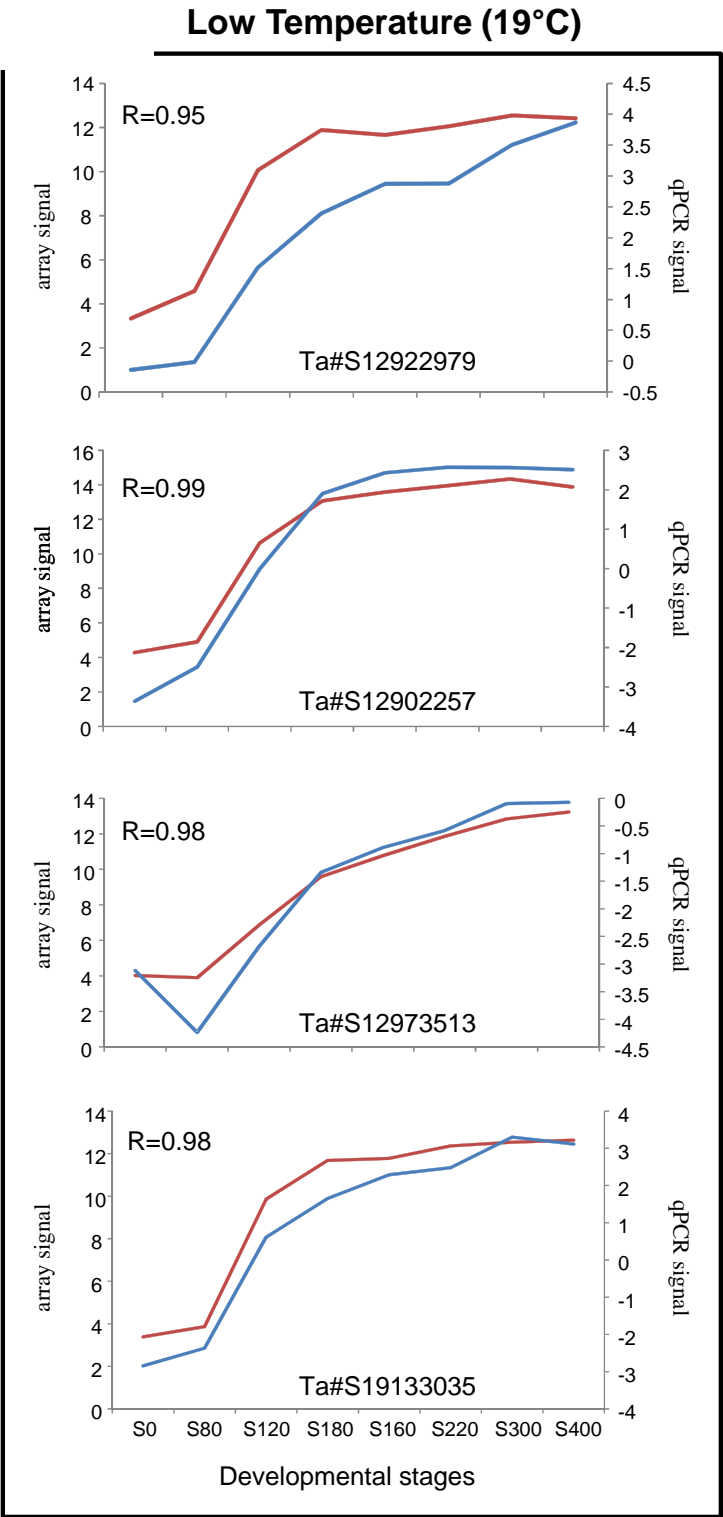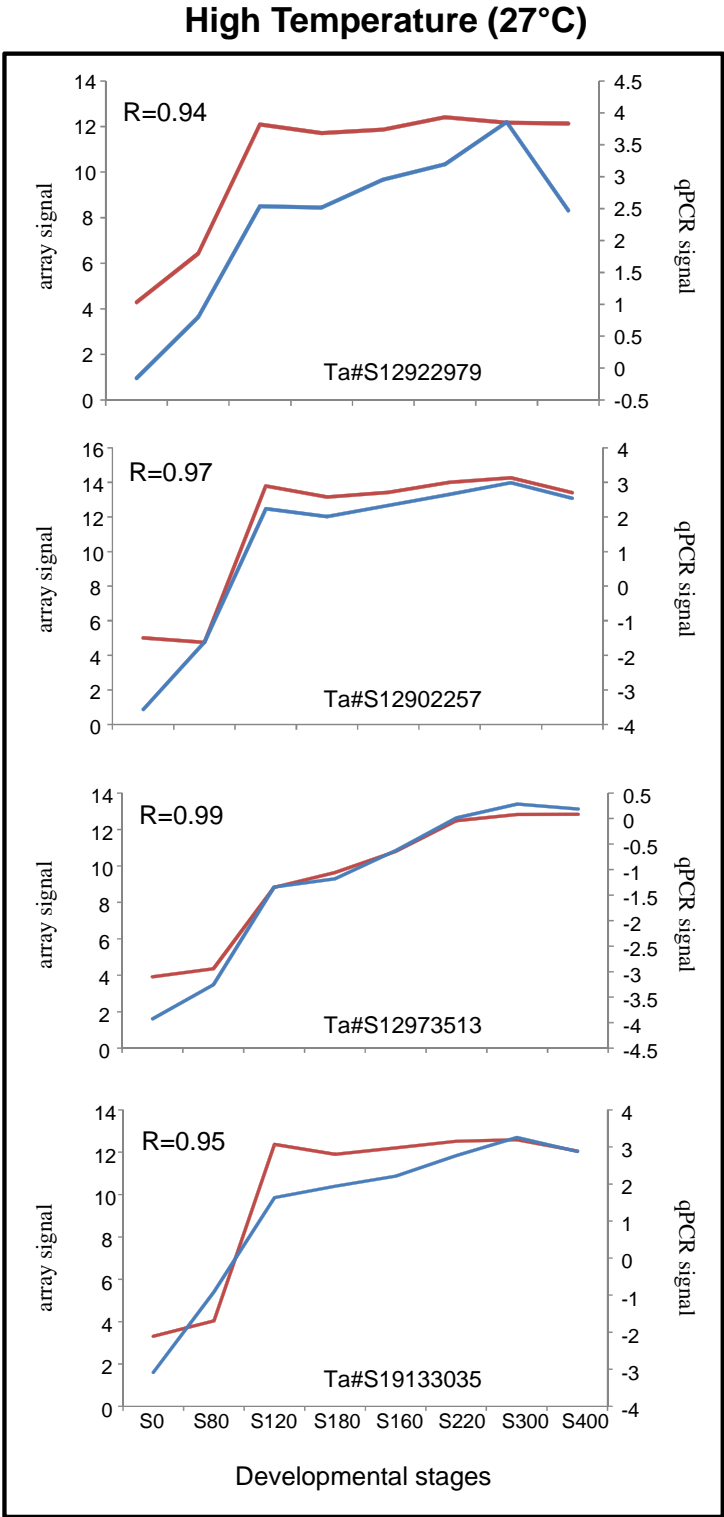

Microarray  
Real-time PCR

Supplement: S2 Fig — Red graphs represent DNA microarray data depicting the expression intensity of each transcript (left y-axis). Blue graphs depict qRT-PCR results (right y-axis representing the relative expression level) during the nine developmental stages (x-axis representing the thermal time after anthesis (°Cdays)). The correlation coefficient (R) between the two graphs is indicated for each gene. (PDF) [file pone.0199434.s002.pdf]

**S3 Fig**

Scale Free Topology Model Fit, signed  $R^2$

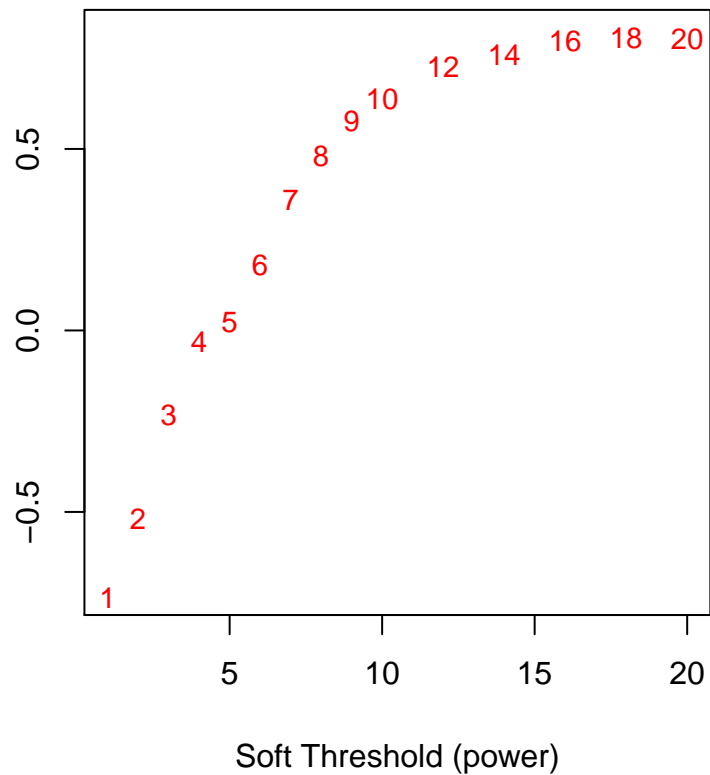

Mean Connectivity

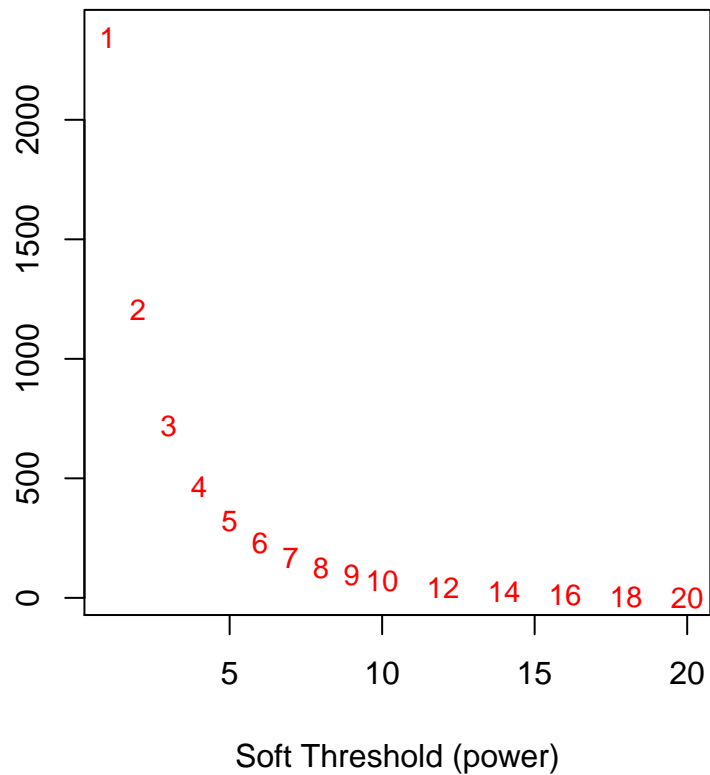

Supplement: S3 Fig — The left panel shows the scale-free fit index (y-axis) as a function of the soft-thresholding power (x-axis). The right panel displays the mean connectivity (degree, y-axis) as a function of the soft-thresholding power (x-axis). (PDF) [file pone.0199434.s003.pdf]

**S4 Fig**

Scale Free Topology Model Fit, signed  $R^2$

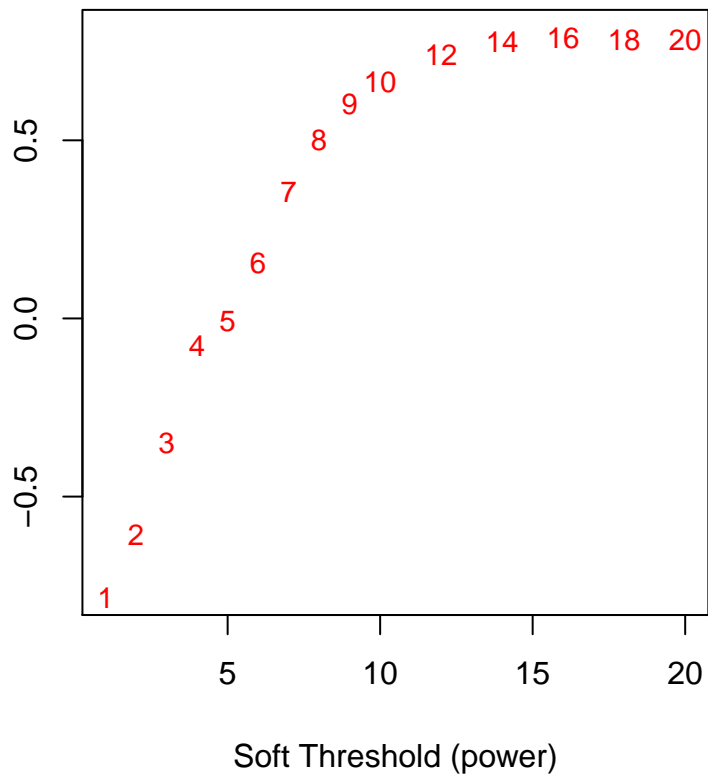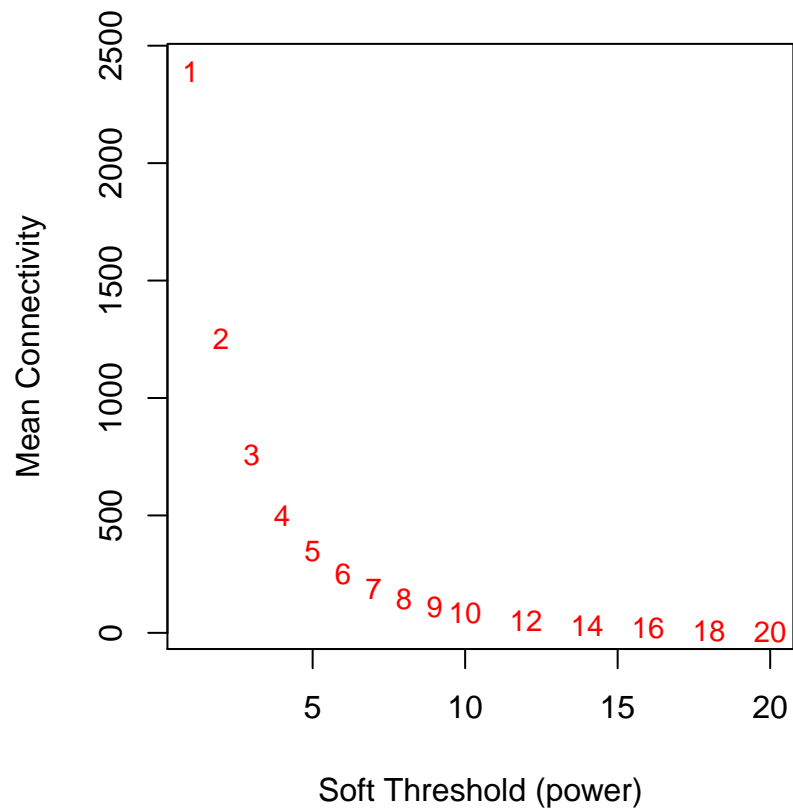

Supplement: S4 Fig — The left panel shows the scale-free fit index (y-axis) as a function of the soft-thresholding power (x-axis). The right panel displays the mean connectivity (degree, y-axis) as a function of the soft-thresholding power (x-axis). (PDF) [file pone.0199434.s004.pdf]

S5 Fig

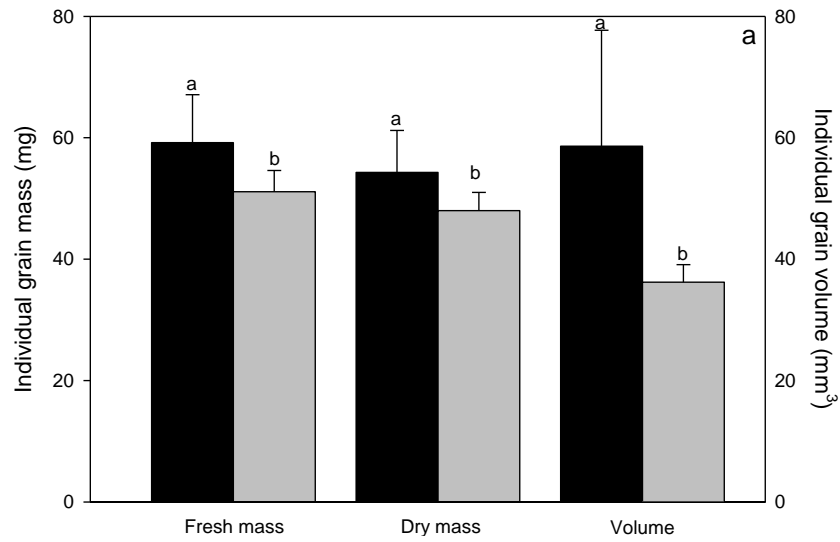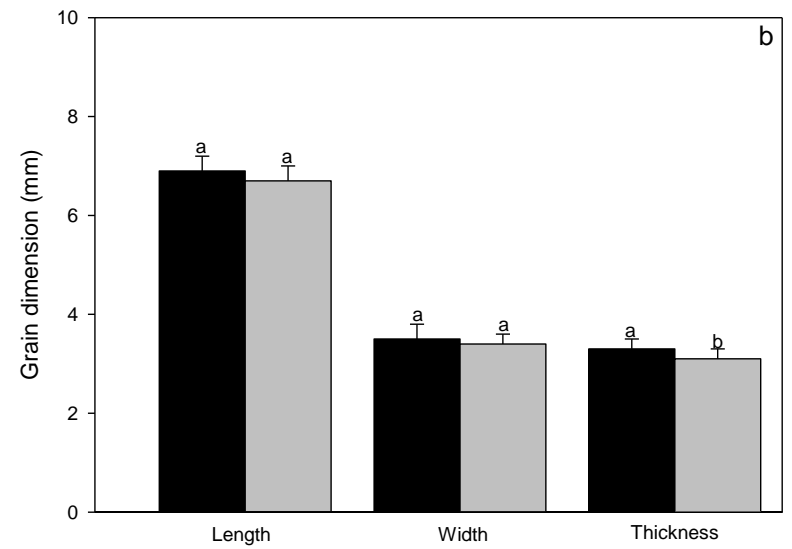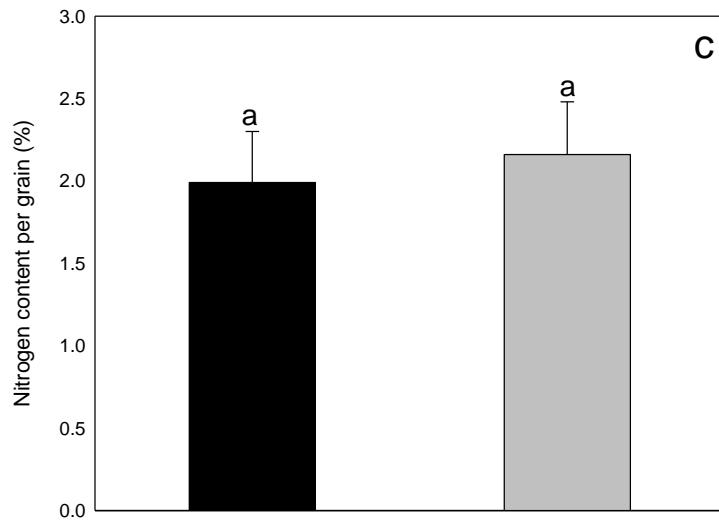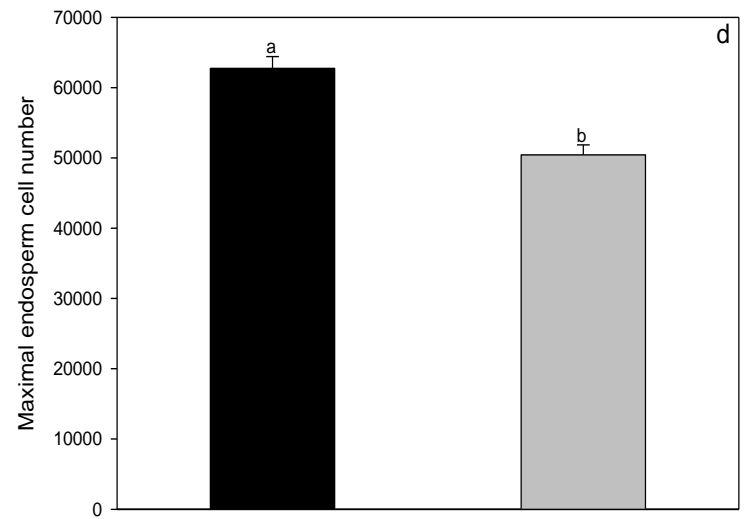

Supplement: S5 Fig — Means (n = 10) differing at a 5% level are indicated by different letters above vertical bars. (PDF) [file pone.0199434.s005.pdf]

S6 Fig

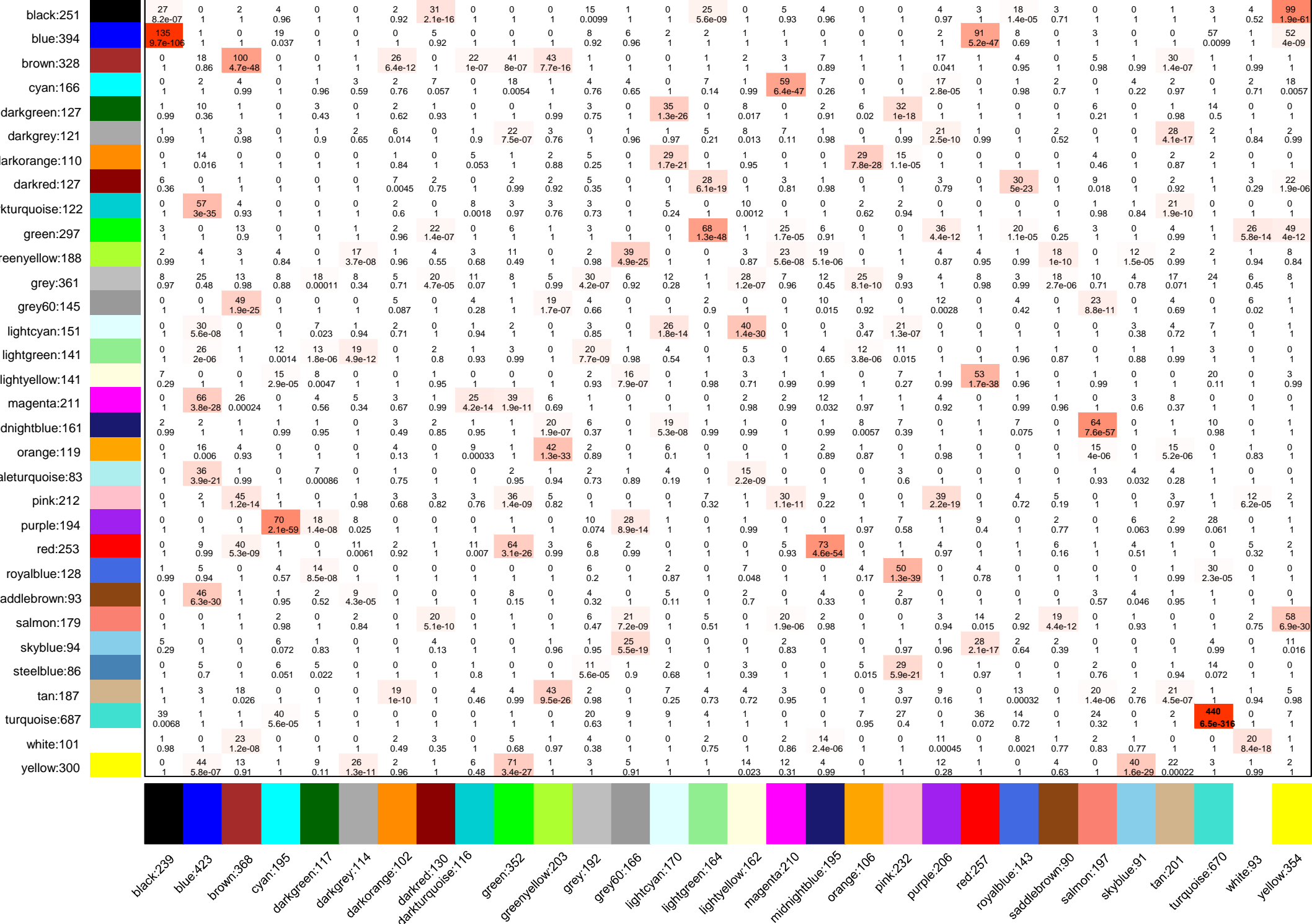

Supplement: S6 Fig — Each row of the table corresponds to one of the 32 modules in the LT network (19°C) and each column corresponds to one of the 30 modules in the HT network (27°C). Numbers indicate gene counts in the intersection of the corresponding modules and the -log(p), with p being the Fisher's exact test p-value for the overlap of the two modules. (PDF) [file pone.0199434.s006.pdf]

**S7 Fig**

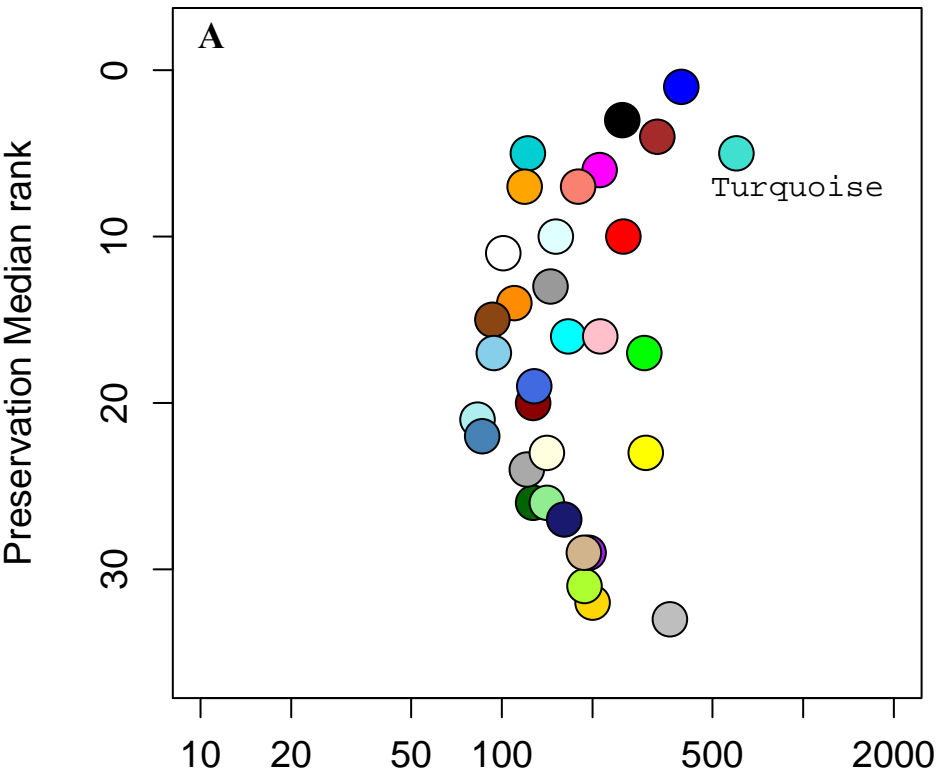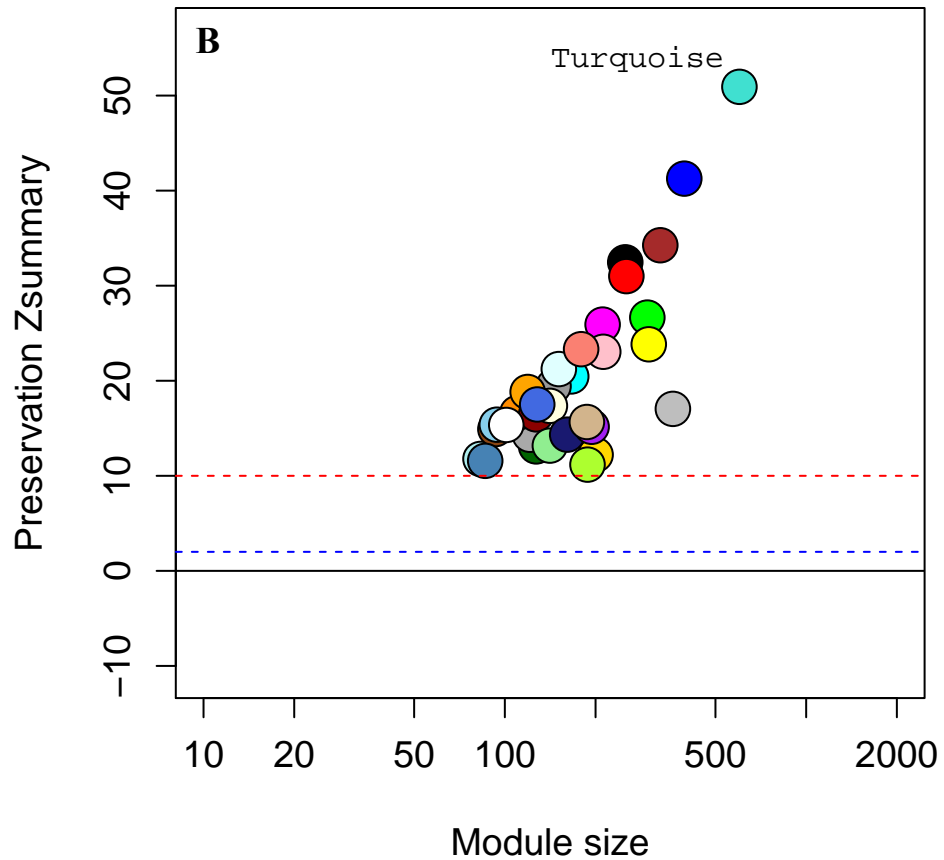

Supplement: S7 Fig — A. The composite statistic medianRank (y-axis) as a function of the module size. Each point represents a module, labeled by color. Low numbers on the y-axis indicate a high preservation. B. The summary statistic Zsummary (y-axis) as a function of the module size. Each point represents a module, labeled by color. The dashed red and blue lines indicate the thresholds Z = 2 and Z = 10, respectively. (PDF) [file pone.0199434.s007.pdf]

S8 Fig

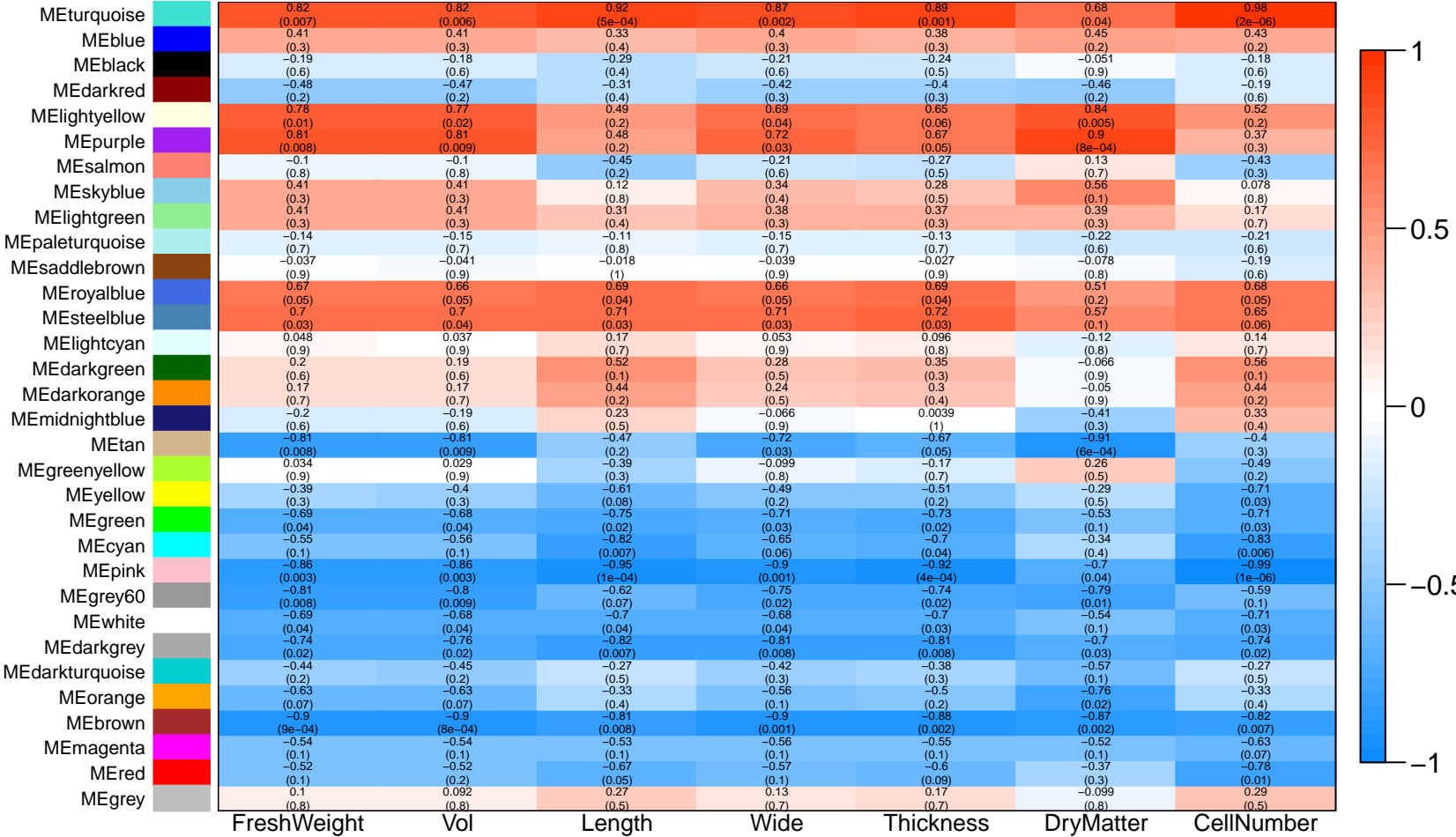

Supplement: S8 Fig — Correlation and p-value are given for each module-trait combination. (PDF) [file pone.0199434.s008.pdf]

S9 Fig

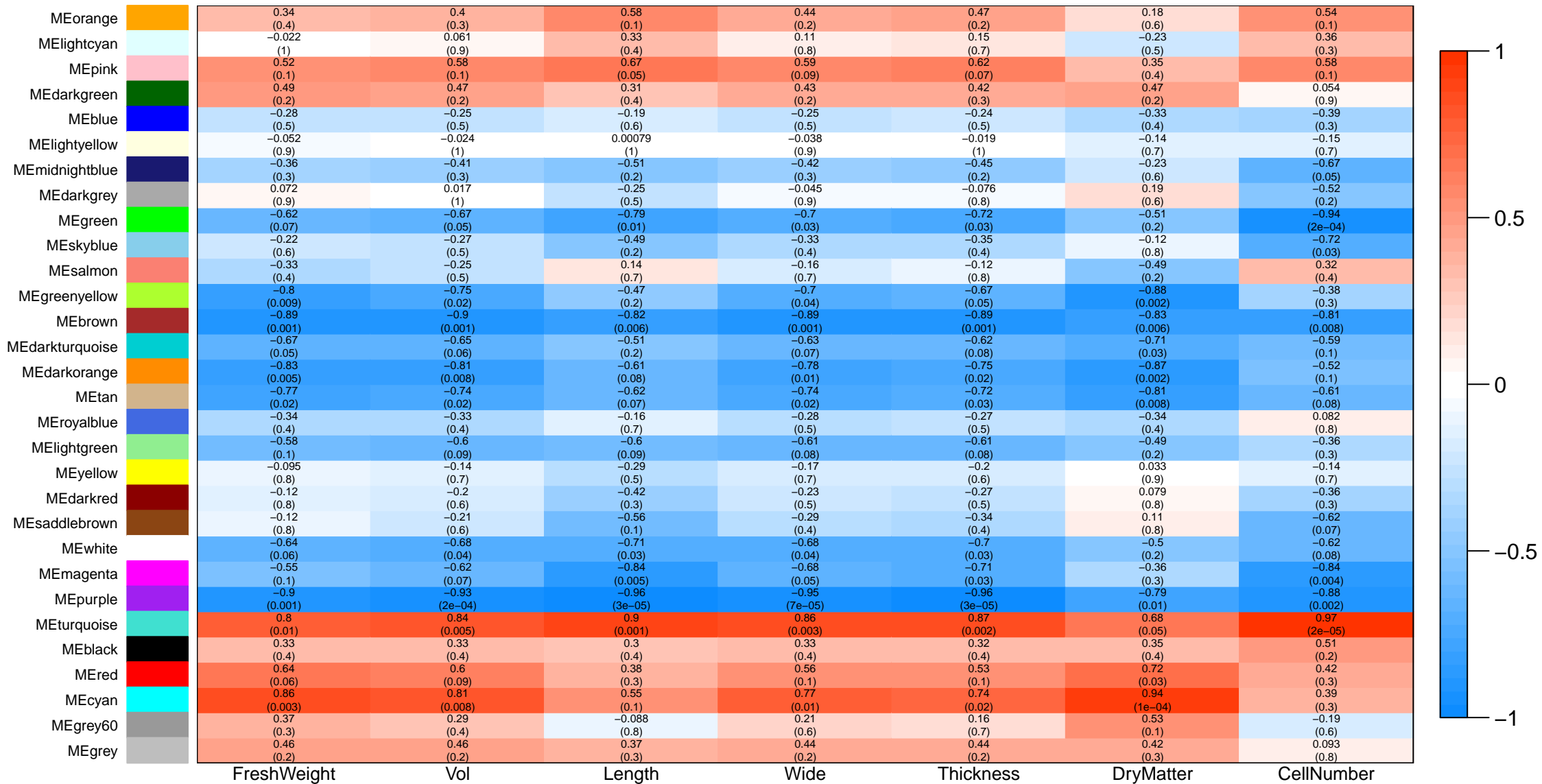

Supplement: S9 Fig — Correlation and p-value are given for each module-trait combination. (PDF) [file pone.0199434.s009.pdf]

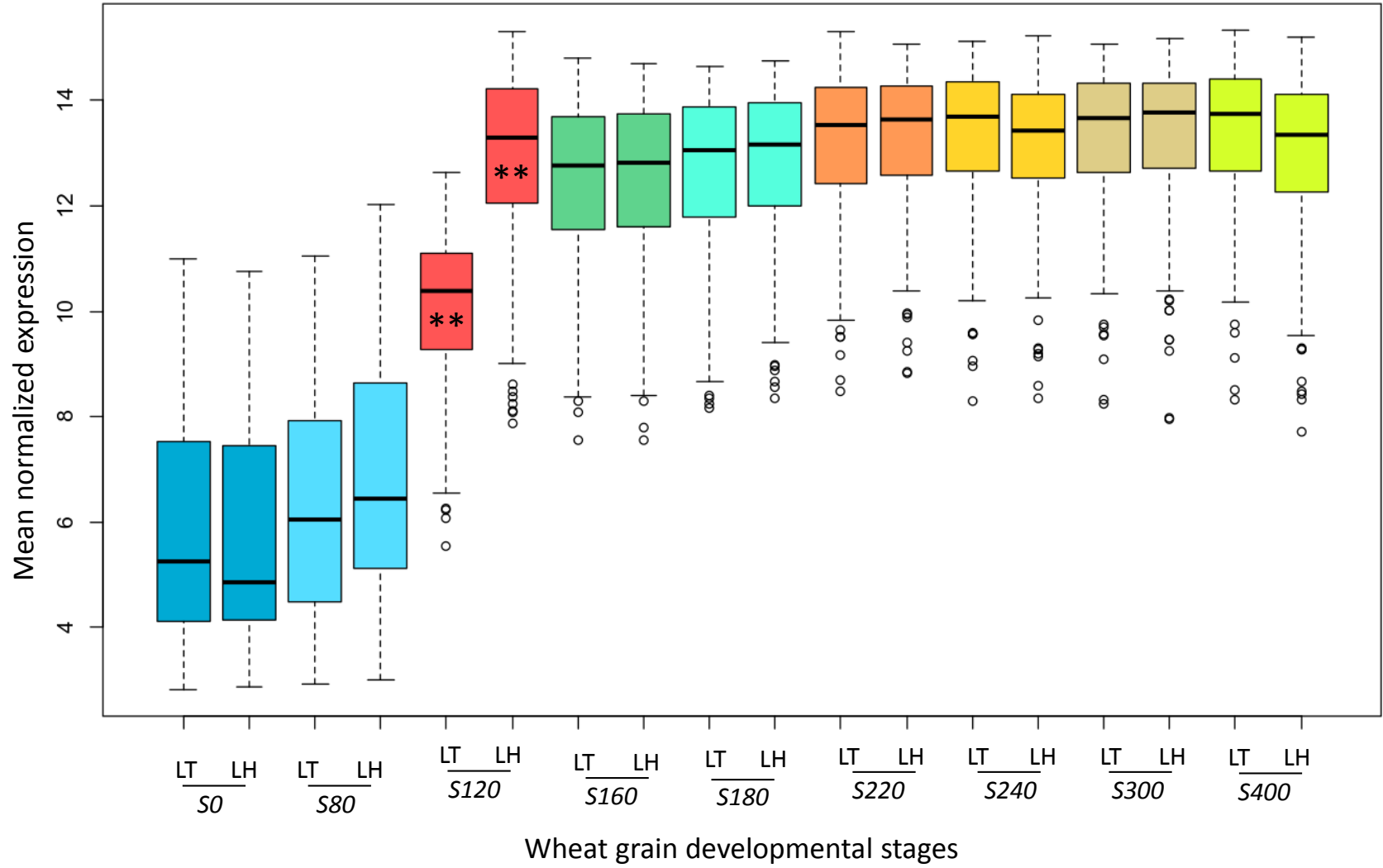

**S10 Fig**

Supplement: S10 Fig — The mean normalized expressions of top 200 genes displaying the greatest Gene Significance of the association with the trait "Cell number in the endosperm" were retrieved and plotted according to wheat grain developmental stages. At each time-point, the mean expression of the genes at 19°C (LT experiment) and at 27°C (HT experiment) were plotted alongside with the standard deviation. Asterisks denote highly significant differences (TukeyHSD test, p<0.001). (PDF) [file pone.0199434.s010.pdf]
